# Innovative multifunctional hybrid photoelectrode design based on a ternary heterojunction with super-enhanced efficiency for artificial photosynthesis

**DOI:** 10.1038/s41598-020-67768-y

**Published:** 2020-06-30

**Authors:** Wayler S. dos Santos, Éder J. Carmo, Yanela Mendez-González, Lucas L. Nascimento, Antônio O. T. Patrocínio, Ruyan Guo, Amar S. Bhalla, Jean-Claude M’Peko, José D. S. Guerra

**Affiliations:** 1 Group of Ferroelectrics and Multifunctional Materials, Institute of Physics, Federal University of Uberlandia, Uberlândia, Minas Gerais 38408-100 Brazil; 2 Physics Faculty/IMRE, University of Havana, 10400 Havana, Cuba; 3 Laboratory of Photochemistry and Materials Science, Institute of Chemistry, Federal University of Uberlandia, Uberlândia, Minas Gerais 38408-100 Brazil; 4 Multifunctional Electronic Materials and Devices Research Lab, Department of Electrical and Computer Engineering, College of Engineering, The University of Texas At San Antonio, San Antonio, TX 78249 USA; 5 São Carlos Institute of Physics, University of São Paulo, São Carlos, São Paulo 13560-970 Brazil

**Keywords:** Environmental sciences, Materials science

## Abstract

Electrochemical cells for direct conversion of solar energy to electricity (or hydrogen) are one of the most sustainable solutions to meet the increasing worldwide energy demands. In this report, a novel and highly-efficient ternary heterojunction-structured Bi_4_O_7_/Bi_3.33_(VO_4_)_2_O_2_/Bi_46_V_8_O_89_ photoelectrode is presented. It is demonstrated that the combination of an inversion layer, induced by holes (or electrons) at the interface of the semiconducting Bi_3.33_(VO_4_)_2_O_2_ and Bi_46_V_8_O_89_ components, and the rectifying contact between the Bi_4_O_7_ and Bi_3.33_(VO_4_)_2_O_2_ phases acting afterward as a conventional *p–n* junction, creates an adjustable virtual *p–n–p* or *n–p–n* junction due to self-polarization in the ion-conducting Bi_46_V_8_O_89_ constituent. This design approach led to anodic and cathodic photocurrent densities of + 38.41 mA cm^–2^ (+ 0.76 V_RHE_) and– 2.48 mA cm^–2^ (0 V_RHE_), respectively. Accordingly, first, this heterojunction can be used either as photoanode or as photocathode with great performance for artificial photosynthesis, noting, second, that the anodic response reveals exceptionally high: more than 300% superior to excellent values previously reported in the literature.

## Introduction

Water splitting by photoelectrochemical cells (PECs) in the presence of light is a sustainable approach to directly convert solar energy into storable chemical energy (H_2_ fuel)^1–5^. Efforts to solve a number of deficiencies and to gain in performance have included modifying the electronic structure of the materials, constructing favorable surface structures with heterojunction, or controlling the morphology^6,7^. The heterojunction concept is actually an excellent alternative for designing materials with improved photocatalytic properties^7^, because promoting a good separation and transport of the photogenerated charges^8,9^. This is the case of bismuth vanadate- and bismuth oxide-based heterojunction systems like BiVO_4_/Bi_4_V_2_O_11_^10–12^, BiVO_4_/Bi_2_O_3_^13^, α-Bi_4_V_2_O_11_/β-Bi_4_V_2_O_11_^14^, Bi_24_O_31_Br_10_/Bi_4_V_2_O_11_^15^, CdS/Bi_4_V_2_O_11_^16^, Bi_2_O_3_/Bi_4_V_2_O_11_^17^, Bi_2_O_3_/BiPO_4_^18^, WO_3_/BiVO_4_/TiO_2_^19^, BiOCl/BiVO_4_/N-GQD^20^, and TiO_2_/BiVO_4_/SnO_2_^21^ that have been tested. Important features such as band-gap energies in the visible spectrum range and the appropriate energy levels of the valence (and conduction) bands make bismuth-vanadium oxides-based compounds attractive to be combined in *p–n* PECs for water splitting^10^.

Overall, in this topic, different design models of PECs such as *p–n* PEC, PEC/photovoltaic, PEC/electroplating devices and others^9,22–24^ have been proposed, with focus always on improving device performance. Among these configurations, the *p–n* PECs formed by a *n*-type semiconductor photoanode and a *p*-type semiconductor photocathode are simpler approaches. The use of PECs with two (or more) light absorbers is advantageous because of the reduction of the thermodynamic potential needed for water splitting into O_2_ (+ 1.23 V_RHE_) and H_2_ (0 V_RHE_)^11,25^. In addition, configuration of the *p–n* PEC also provides greater output photovoltage to form the H_2_ and O_2_ products from water splitting^5^. In order to successfully perform the photoelectrochemical decomposition of water without the need of applied potentials, we just note that the photovoltage of the cell must exceed + 1.23 V under light irradiation^11^.

With eyes on the bismuth- and vanadium-based materials, a multifunctional photoelectrode based on the unprecedented combination of Bi_46_V_8_O_89_, Bi_3.33_(VO_4_)_2_O_2_ and Bi_4_O_7_ was designed and constructed in this work. Bi_46_V_8_O_89_ is an ionic conductor with a remarkable conductivity, where oxygen ion migration pathways include not only the diffusion of the vacancy through the Bi–O sub-network, commonly found in δ-Bi_2_O_3_ based superstructures, but also the exchange of O^2–^ between the Bi–O and V–O sub-networks^26,27^. Bi_3.33_(VO_4_)_2_O_2_ is a semiconductor-like material with an optical band-gap around 2.36 eV, and exhibits a great photocatalytic behavior for the decomposition of, e.g., phenol under visible light irradiation^28^. Bi_4_O_7_ is also a semiconductor-like material, exhibiting a strong visible light absorption with an edge around 700 nm^29^. The energies of the conduction and valence bands were in this material estimated to be about 0.63 eV and 2.52 eV versus NHE (Normal Hydrogen Electrode), giving a band-gap value of about 1.89 eV^30^. We show that when the Bi_46_V_8_O_89_ ionic-conductor is coupled to Bi_4_O_7_/Bi_3.33_(VO_4_)_2_O_2_, its spontaneous-like electrical polarization generates an electric field high enough to produce an inversion layer at the interface, ending with an increased efficiency for generation, transport and separation of the photogenerated charges. The consequence is observation of a super-enhanced photoelectrochemical activity due to the right combination of a rectifier contact and an inversion layer, enabling an adequate alignment of the energy bands. In addition, we demonstrate that nature of conductivity in this photoelectrode heterostructure can be also adjusted in a tuning-like way depending on the applied potential and frequency, the consequence of which is also discussed.

## Results

### Photoelectrode synthesis and characterization

**Figure 1:**
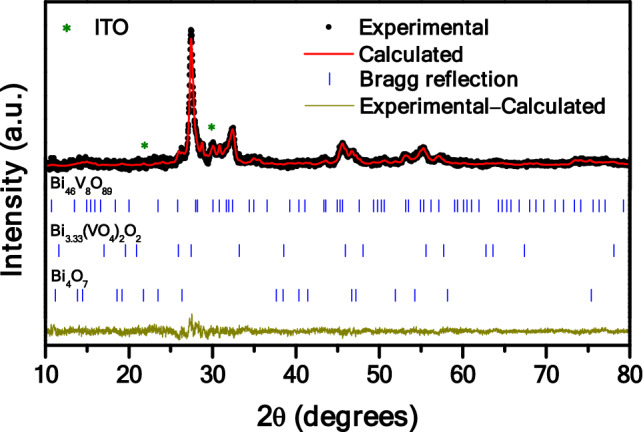
XRD pattern and Rietveld refinement of the prepared BVO film.

One of the components targeted in this work is Bi_4_O_7_, and its synthesis on a large scale and high-purity grade is known to be a challenge. But, also known is that this oxide usually appears as an impurity during processing of other bismuth oxides^29^, and this was the approach considered here through controlling synthesis parameters. Briefly, particulate composite-like bismuth- and vanadium-containing photoelectrode films, simply abbreviated as BVO, were produced from high-purity Bi_5_O(OH)_9_(NO_3_)_4_ and NH_4_VO_3_ reagents, followed by deposition on transparent conducting indium tin oxide (ITO) coated glass, as described in the Experimental section. The films were characterized in terms of X-ray diffraction (XRD), Raman spectroscopy (RS), scanning electron microscopy (SEM), and diffuse reflectance spectra (DRS). Figure 1 shows the XRD pattern from the prepared BVO material, the data of which reveals presence of the three phases intended in this work: Bi_46_V_8_O_89_ (ICSD 415113), Bi_3.33_(VO_4_)_2_O_2_ (COD 1507777) and Bi_4_O_7_ (ICSD 51778). Indeed, getting the two former phases involved a careful control of the synthesis process, which showed a clear influence on the stoichiometry of the end product. That is, by using acetic acid (pH ≈ 2), formation of the Bi_46_V_8_O_89_ phase, which is self-doped with Bi^5+^, was verified. Furthermore, when ammonium hydroxide was added to change the pH to 8, a decomposition of part of Bi_46_V_8_O_89_ into Bi_4_V_2_O_11_ was favored. Besides these bismuth- and vanadium-containing photoactive phases, diffraction peaks corresponding to the ITO substrate were also identified in Fig. 1 (ICSD 85084). Refinement of the XRD data by applying the Rietveld method allowed assessing the crystallographic parameters of these phases, and the results are presented in Table S1, remaining totally comparable with data from the literature^26,28,31^.

**Figure 2:**
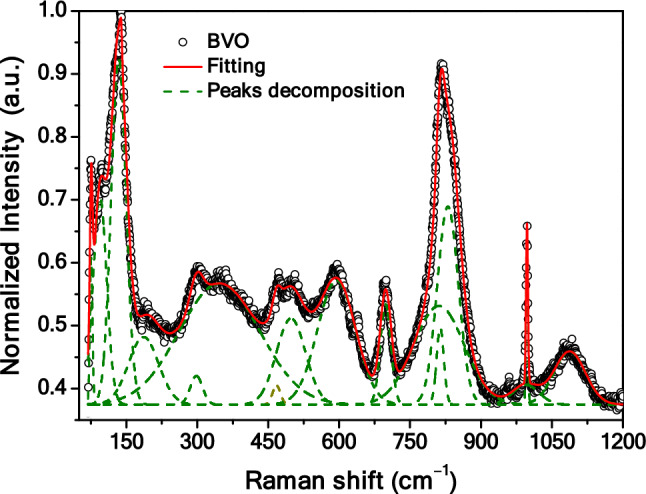
Measured Raman scattering spectrum (open symbols), together with the fitted spectrum (solid line) and the decomposed active modes (dashed lines) of the BVO film.

In order to get additional information about the BVO ternary heterostructure, Raman spectroscopy was performed, and the result is shown in Fig. 2. The data was fitted using a theoretical method based on the location of the curvature maxima in concave-down (CMCD) spectral regions of the recorded Raman spectrum^32^. The fitting curve of the measured spectrum (solid line) as well as the decomposed contributions into individual Lorentzian components (dashed lines) are also shown in Fig. 2. The peaks position for each Raman active mode (natural wavenumber, cm^–1^), obtained by the aforementioned CMCD method, and the assigned vibrational modes are summarized in Table S2. The peaks with long correlation-lengths found around 498.08 and 594.51 cm^–1^ are assigned to the Bi–O (OBi_4_) vibrational mode, while those with short correlation-lengths observed at 75.61, 94.03, 133.63, 186.31 and 298.12 cm^–1^ are assigned to the Bi–O (OBi_3_) vibrational mode^33^. In particular, the peak at 94.03 cm^–1^ is caused by the Bi^(3–x)+^ and Bi^5+^ species^34^. The bands at 347.02 cm^–1^ and 467.43 cm^–1^ are assigned to the ν_2_ and ν_4_ symmetric flexion modes of the VO_4_ tetrahedron, respectively^33,35,36^. The peak located at 698.17 cm^–1^ is attributed to the ν_3_ anti-symmetric mode of the VO_4_ tetrahedron, while the peaks at 810.96, 813.74 and 829.83 cm^–1^ are assigned to the ν_1_ symmetric mode of the V–O bond^33,35,36^. The Raman band located at 640.64 cm^–1^ is related to the double-coordinated oxygen atom of the V–O–V bond^33,35^. Finally, bands related to the vanadyl oxygen expansion mode V^4+^ = O, located at 994.67 and 997.29 cm^–1^, and V^5+^ = O, located at 1,087.47 cm^–1^, were observed^37^. The presence of these bands suggests the existence of the two bismuth oxide vanadate phases, which is a consequence of the OH^–^ action in the aqueous solution during synthesis control, as we mentioned above.

The following reaction (a) represents the synthesis process under acidic pH conditions, using acetic acid as a solvent for the reagent dilution.

- $$46{\mathrm{Bi}}_{5}{\left(\mathrm{OH}\right)}_{9}{\left(\mathrm{NO}_{3}\right)}_{4}+40{\mathrm{NH}}_{4}\mathrm{VO}_{3}\to 5{\mathrm{Bi}}_{46}{\mathrm{V}}_{8}{\mathrm{O}}_{89}+177{\mathrm{O}}_{2}+112{\mathrm{N}}_{2}+287{\mathrm{H}}_{2}\mathrm{O}$$

The Bi_5_O(OH)_9_(NO_3_)_4_ compound is formed after mixing of bismuth nitrate and bismuth oxide, having the used reagent in the present work about 79% of Bi_2_O_3_ dosage. After reacting with NH_4_VO_3_, it would result in the formation of Bi_46_V_8_O_89_. However, for a conversion of ~ 100% of Bi_46_V_8_O_89_, as described in the previous reaction, the crystallization process would have to be necessarily performed at higher temperatures (≥ 700 °C)^26^; nevertheless, as the correction was carried out under a basic pH condition, according to the reactions (b) and (c), and the crystallization of the BVO film was performed at 450 °C, it was already expected that other crystalline phases would be formed during the thermal decomposition^26,27^. Furthermore, it is known that corner sharing ${\mathrm{BiO}}_{3}^{-}$ e $\mathrm{VO}_{3}^{-}$ ions could give rise to mixed oxides^38–40^ (Bi_3.33_(VO_4_)_2_O_2_ and Bi_4_O_7_) by the action of hydroxyl radicals from the NH_4_OH base used in the synthesis process, as can be seen by the following reactions (b) and (c):

(b) $$4\mathrm{BiO}_{3}^{-}+2\mathrm{OH}^{-}\to {\mathrm{Bi}}_{4}{\mathrm{O}}_{7}+3{\mathrm{O}}_{2}+{\mathrm{H}}_{2}\mathrm{O}$$
(c) $$3.33\mathrm{BiO}_{3}^{-}+2{\mathrm{VO}}_{3}^{-}+\mathrm{OH}^{-}\to {\mathrm{Bi}}_{3.33}{\left(\mathrm{VO}_{4}\right)}_{2}{\mathrm{O}}_{2}+3.245{\mathrm{O}}_{2}+0.5{\mathrm{H}}_{2}\mathrm{O}$$

After such reactions, citric acid was used in order to stabilize the bismuth metallic ions during the drying process. On the other hand, in order to provide additional support for the good correspondence between the observed results from XRD data and Raman spectroscopy, complementary structural analysis has been considered, which involves two fundamental parameters that can be used to get further structural information regarding the synthesized phases, as is the case of the average crystallite size (D) and structural micro-strain (ϵ). Both parameters were estimated from the XRD pattern using the Williamson–Hall equation:^41,42^

1 $${\upbeta }_{\mathrm{hkl}}\cos \uptheta =\frac{{\mathrm{K}}\uplambda }{{\mathrm{D}}}+4\upepsilon \sin\uptheta $$

where K is a proportionality constant related to the particle geometry, λ is the wavelength, θ is the Bragg’s angle and β_hkl_ represents the full-width at half-maximum of the diffraction peaks. The results are shown in Table 1.

**Table 1:** Average crystallite size (D) and structural micro-strain (ϵ) obtained for the film BVO, by using the Williamson–Hall equation.

Parameters	Crystalline phases		
	Bi_46_V_8_O_89_	Bi_3.33_(VO_4_)_2_O_2_	Bi_4_O_7_
D (10^–9^ m)	38.08	25.66	33.15
ϵ (10^–4^ m)	4.87	– 2.28	3.40

As can be seen, while a positive strain for Bi_46_V_8_O_89_ and Bi_4_O_7_ phases has been observed, a structural self-doping with Bi^5+^ ions, with ionic radius around 76 pm (or 0.76 Å), causes a negative strain for the Bi_3.33_(VO_4_)_2_O_2_ phase. This leads to an increase of the unit-cell volume in Bi_46_V_8_O_89_ and Bi_4_O_7_ and a decrease of volume for Bi_3.33_(VO_4_)_2_O_2_, the consequence of which is a reduced average crystallite size for the latter. Due to the bismuth deficiency, the lattice oxygen ions (O^2–^) undergo a charge transfer with the Bi^3+^ ions on the surface of the BVO heterostructure. Therefore, due to the ions’ mobility, we consider that there is a double valence fluctuation scheme involving the change of two electrons on the surface. The double valence variation process, as described in the following reaction (c), occurs due to the dimerization of the ligand holes (*h*^+^) caused by direct ligand–ligand hybridization, which is expected in perovskite-type structures in which there is an oxygen accumulation nearby. This process occurs, in particular, when the spin density of Bi^3+^ is small and it participates in the double valence fluctuation.

(c) $${\mathrm{Bi}}^{3+}+2{\mathrm{O}}^{2-}\to {\mathrm{Bi}}^{5+}+{\mathrm{O}}_{2}^{2-}$$

Under thermal evacuation, the bismuth oxide with the Bi^3+^ (6p°) electronic configuration is transformed into its Bi^5+^ (6s°) state by double valence flotation^43,44^. For complementary information, SEM image of the films is presented in Figure S1, the result of which revealed the BVO material to consist of islands of self-assembled nanoplates, forming nano-porous sponges. In addition, the cross-sectional view, shown in Figure S2, indicated to be dealing with films showing a thickness of about ~ 10 m.

On the other hand, the DRS data represented through the transmittance (T) and reflectance (R) spectra, whose results are shown in Figure S3a–b, revealed that the displacement of the bands occurred after the deposition of the BVO film on the ITO. The BVO film was quite rough on the surface due to the higher reflectance, compared to the ITO glass. According to the literature, and some data collected in our Laboratory, values of the direct band-gap energies for the model (individual) compounds are: 1.89 eV for Bi_4_O_7_, 2.36 eV for Bi_3.33_(VO_4_)_2_O_2_ and 2.93 eV for Bi_46_V_8_O_89_^27,28,30^. These values are comparable to those estimated in this work from Figure S3c, except for Bi_4_O_7_, where the synthesis procedure was determinant in reducing its value. In any case, these results indicate that all the three components in the BVO heterojunction can be individually excited by visible light to produce the reactive species, that is to say, electrons and holes needed to trigger water splitting.

**Figure 3:**
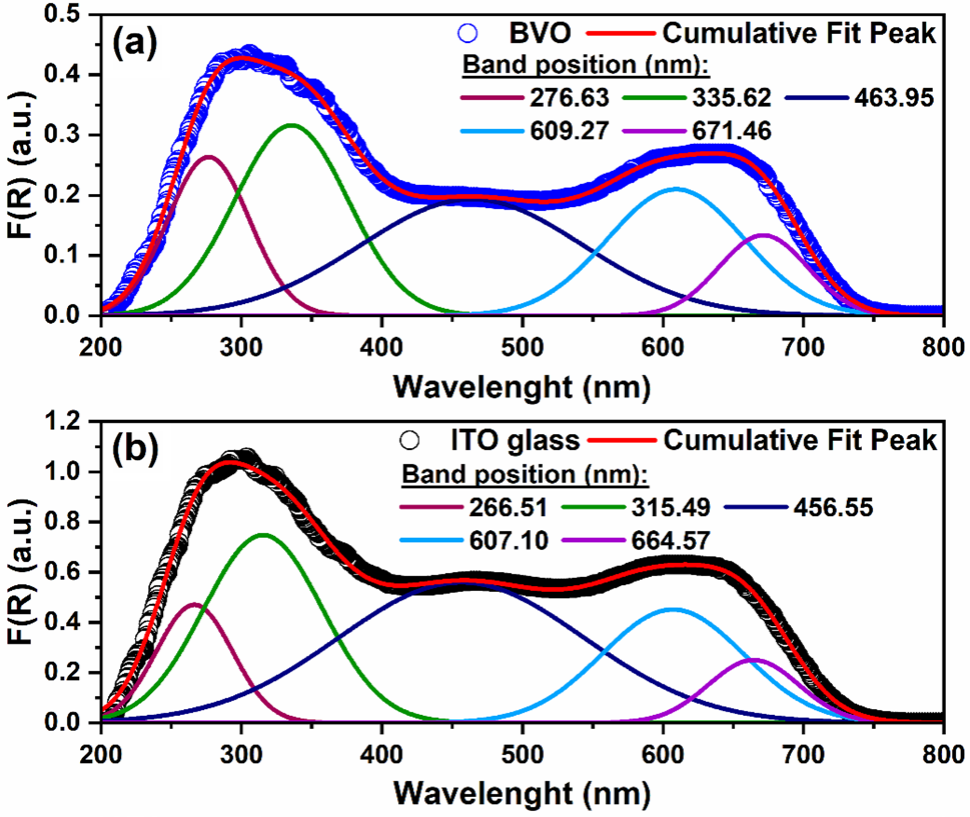
Kubelka–Munk absorbance: (**a**) BVO film and (**b**) ITO glass.

Regarding the main interest spectrum, i.e. Kubelka–Munk absorbance (F(R)), we proceeded with a Gaussian deconvolution of the absorption bands, the results of which are shown in Fig. 3. Five absorption bands were identified, which presented wavelength displacements towards the region of the visible spectrum for the BVO film. Such a displacement is caused by defects in the crystalline structure of the BVO, and it is expected to promote donor (or acceptor) states between the energy bands, giving rise to certain charge trap states between the bands. The charge carriers trapping in these states could play a crucial role in the optical and photoelectrochemical properties of the BVO film^45^.

**Figure 4:**
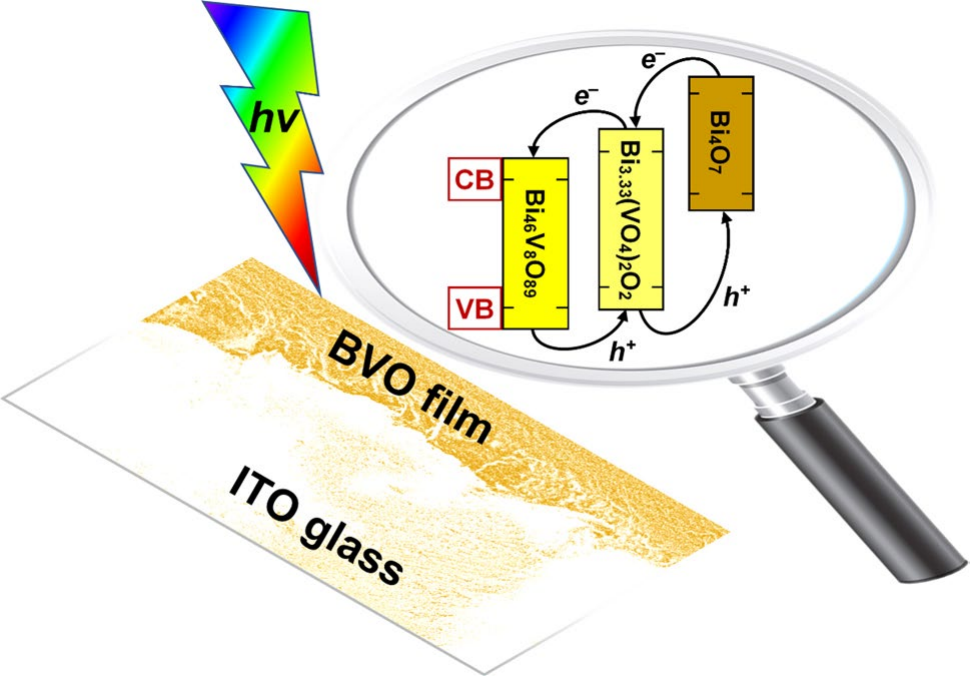
Alignment of conduction (CB) and valence (VB) bands for the Bi_4_O_7_–Bi_3.33_(VO_4_)_2_O_2_–Bi_46_V_8_O_89_ (abbreviated as BVO) particulate composite-like ternary heterojunction.

The adequate alignment of the conduction (CB) and valence (VB) bands applying in this situation is briefly outlined in Fig. 4 (with additional details given later).

The results presented in the following refer to data extracted from Mott–Schottky, photoelectrochemical performance and impedance spectroscopy measurements, aimed at getting insights into the materials chemistry-controlled electrical and electrochemical responses from these heterojunction-structured BVO films. All these experiments were conducted using a standard three-electrode cell configuration^46^, with an Ag/AgCl (3.0 M KCl) reference electrode, a platinum metalized FTO substrate as the counter electrode, and the BVO film acting as the working electrode (as described in Methods). For the sake of a direct comparison, the electrochemical performance and impedance data were collected under both dark and illumination conditions^46^.

### Mott–Schottky data

The conductivity characteristics of the heterojunction-structured BVO material were partly elucidated by the Mott–Schottky electrochemical characterization, and interpreted through the mathematical model described by the equation below:

2 $${\mathrm{Cp}}^{-2}=\left(\frac{2}{\varepsilon{\varepsilon }_{0 }{A}^{2} e {\mathrm{N}}_{\mathrm{D}}}\right)\left(\mathrm{V}-{\mathrm{V}}_{\mathrm{fb}}-\frac{{\mathrm{k}}_{\mathrm{B}}T}{e}\right)$$

from which a linear behavior between the inverse of the square capacitance (Cp^–2^) of the space-charge layer and the applied potential (V) is predicted. In this expression, N_D_ is the number of donor ions, k_B_ the Boltzmann’s constant, ε the dielectric constant of the semiconductor, *A* the area of the electrode, ε_0_ the dielectric permittivity of free space, *T* the absolute temperature, *e* the electronic charge and V_fb_ the flat-band potential^47,48^.

**Figure 5:**
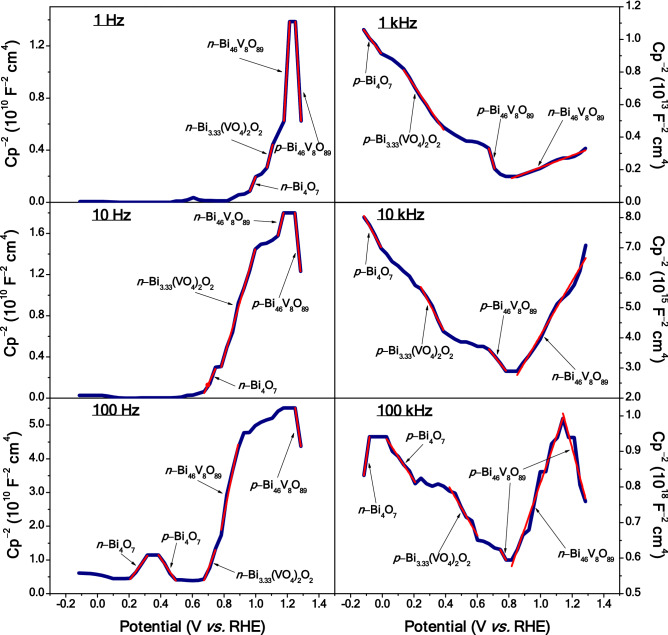
Mott–Schottky plots. Variation of capacitance (C) with the applied potential in a Na_2_SO_4_ aqueous solution (with 0.5 M and pH = 6.6), as extracted from the Mott–Schottky experiments conducted on the BVO film at different frequencies.

The Mott–Schottky results from the BVO film are illustrated in Fig. 5, and the nature (*n* or *p*) of conductivity from each component is there identified. The data reveal that, at low frequencies, in the range of 1–100 Hz (Fig. 4, left-hand side), the photoelectrode mostly shows *n*-type conductivity (straight lines with positive slope) for potential values <  + 1.2 V_RHE_. For values >  + 1.2 V_RHE_, however, an inversion of polarity is observed, which is related to the contact of *n*-Bi_4_O_7_, *n*-Bi_3.33_(VO_4_)_2_O_2_ and *n*-Bi_46_V_8_O_89_, resulting in the formation of a spatial-charge region at the Bi_3.33_(VO_4_)_2_O_2_/Bi_46_V_8_O_89_ interface of this minority *h*^+^-charged junction so as reversing the conductivity of Bi_46_V_8_O_89_ to *p*-type. For high frequencies, in the range of 1–100 kHz (Fig. 4, right-hand side), a modification to mostly *p*-type conductivity occurs (straight lines with negative slope) towards low potential values, with a polarity inversion occurring at + 0.8 V_RHE_ while increasing the potential. In this case, the inversion layer acts in the opposite way, because when the *p*-Bi_46_V_8_O_89_ structure is in contact with the *p*-Bi_3.33_(VO_4_)_2_O_2_ one, the spatial charge region at the interface of the junction will be charged with a minority of negative (*e*^*–*^) charges, and the conductivity of Bi_46_V_8_O_89_ will be of *n*-type.

Regarding particularly Bi_4_O_7_, an additional relevant information seen in this Figure is the polarity change this component shows at 100 Hz and 100 kHz, indicating formation of a dipole moment at the Bi_4_O_7_/Bi_3.33_(VO_4_)_2_O_2_ interface, with apparently a minority of positive (*h*^+^) charges at 100 Hz, and a minority of negative (*e*^*–*^) charges at 100 kHz. According to the literature, the inversion layer cannot be detected for thin films with a single semiconductor^11^. This suggests that this process occurs at the interface between the materials constituting the heterojunction, revealing that in the inversion layer the BVO film may act as a photocathode. For this reason, besides the originally-planned tests for photoanode applications (the results of which are presented in the next section), behavior of the Bi_4_O_7_/Bi_3.33_(VO_4_)_2_O_2_/Bi_46_V_8_O_89_ heterojunction as photocathode was also checked and analyzed through current-potential curves (Figure S4). The BVO heterostructure exhibited a notable photocurrent typical of *p*-type semiconductors at cathodic potential, with a density of– 2.48 mA cm^–2^ at 0 V_RHE_, proving effectively photoactivity of this heterojunction structure as photocathode.

From the application of the Mott–Schottky mathematical model, the conduction energy bands (CB) can be measured as the flat-band potential (V_fb_) and, as a consequence, the valence energy bands (VB) position was calculated by summing the optical band-gap energy and V_fb_ for each component. The CB values obtained at different frequencies for the Bi_4_O_7_, Bi_3.33_(VO_4_)_2_O_2_ and Bi_46_V_8_O_89_ phases were, respectively: 1 Hz (+ 0.94, + 1.02 and + 1.15 V_RHE_), 10 Hz (+ 0.66, + 0.73 and + 0.89 V_RHE_), 100 Hz (+ 0.14, + 0.64 and + 0.70 V_RHE_), 1 kHz (+ 0.69, + 0.69 and + 0.77 V_RHE_), 10 kHz (+ 0.72, + 0.81 and + 1.22 V_RHE_) and 100 kHz (+ 1.41, + 1.47 and + 1.50 V_RHE_). On the other hand, the obtained VB values for Bi_4_O_7_, Bi_3.33_(VO_4_)_2_O_2_ and Bi_46_V_8_O_89_ are, respectively: at 1 Hz (+ 1.73, + 3.33 and + 3.81 V_RHE_), 10 Hz (+ 1.45, + 3.04 and + 3.55 V_RHE_), 100 Hz (+ 0.93, + 2.95 and + 3.36 V_RHE_), 1 kHz (+ 1.48, + 3.00 and + 3.43 V_RHE_), 10 kHz (+ 1.51, + 3.12 and + 3.88 V_RHE_) and 100 kHz (+ 2.20, + 3.78 and + 4.16 V_RHE_). Based on these values, we designed the potential energy diagram showing the alignment of the CB and VB bands for the three Bi_4_O_7_, Bi_3.33_(VO_4_)_2_O_2_ and Bi_46_V_8_O_89_ phases and the figure will be shown later, with an additional discussion. The CB and VB for the Bi_4_O_7_ phase are more negative than the corresponding bands for Bi_3.33_(VO_4_)_2_O_2_, while the CB and VB of Bi_3.33_(VO_4_)_2_O_2_ are more negative than the corresponding bands of Bi_46_V_8_O_89_; this observed behavior favors the easy injection of electrons from the CB of Bi_4_O_7_ to that of Bi_3.33_(VO_4_)_2_O_2_ and CB of Bi_3.33_(VO_4_)_2_O_2_ to that of Bi_46_V_8_O_89_ in the heterostructure.

### Photoelectrochemical performance and impedance spectroscopy

**Figure 6:**
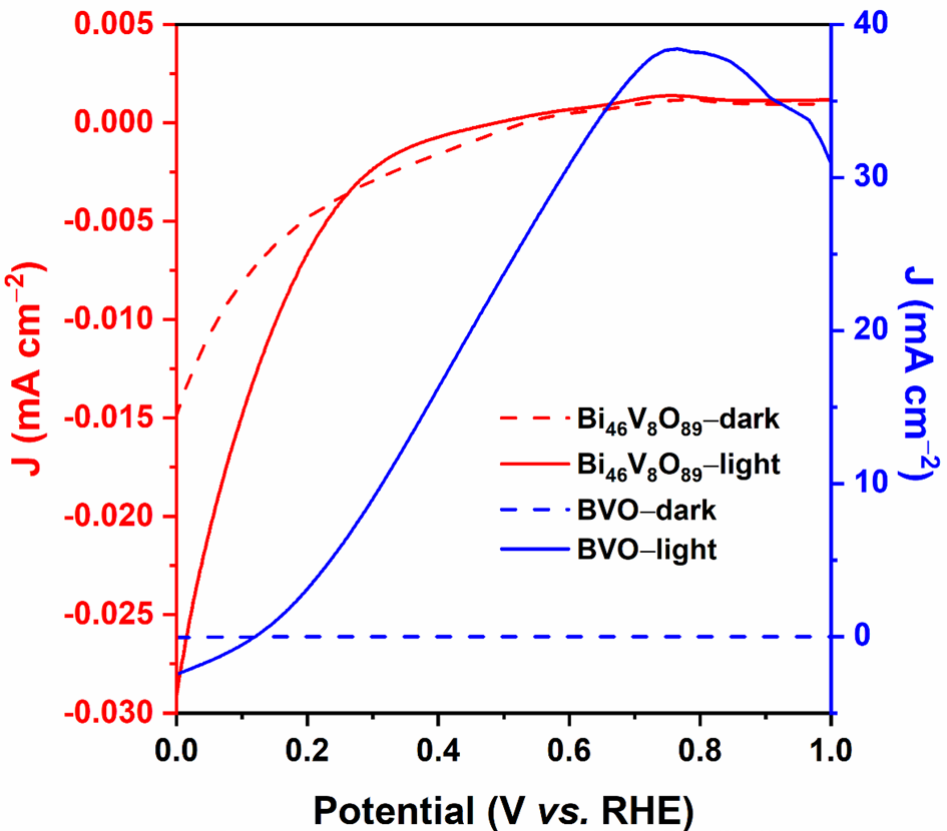
Photoelectrochemical characteristic of the photoelectrode. Current-potential curves for the prepared BVO film, acting as photoanode. Measurement conditions: active area of 0.2 cm^2^, and 0.5 M Na_2_SO_4_ electrolyte (pH = 6.6). Light Source: Xe Lamp (λ > 450 nm, 100 mW cm^−2^), scan rate of 20 mV s^−1^ from low to high potential, in back illumination mode.

The electrochemical performance characteristics of the BVO electrode, tested as photoanode, under dark and illumination conditions are depicted in Fig. 6. Photoactivity of this working electrode is clear, noting that, under light irradiation, the extracted data of anodic (peak value) photocurrent density is + 38.41 mA cm^–2^ (+ 0.76 V_RHE_). Furthermore, the photocurrent density values obtained for the BVO heterostructure were significantly higher than the values for Bi_46_V_8_O_89_, indicating that the formation of the heterojunction improves the charges separation and transport processes. Notice that the result for water oxidation is actually telling, as revealing to be 393% greater than that reported, for instance, for one of the best photoanodes based on parent W–BiVO_4_/V_2_O_5_ films, which achieved a high but comparatively-quite lower photocurrent density of + 7.79 mA cm^–2^ (+ 1.23 V_RHE_)^49^.

These results, showing photoactivity of the BVO film either as photocathode or as photoanode are outstanding because revealing the bifunctionality of this heterojunction-structured photoelectrode for water splitting into H_2_ and O_2_. In addition, the highest (peak) value we obtained for the HC–STH (half-cell solar-to-hydrogen efficiency) of the BVO film was about 19.75% at + 0.65 V_RHE_ (Figure S5). All these results demonstrate that the photoelectrode we built here can promote the photoelectrochemical oxidation of water in O_2_, and is also photoactive in the reduction reaction of water to produce renewable H_2_ fuel.

**Figure 7:**
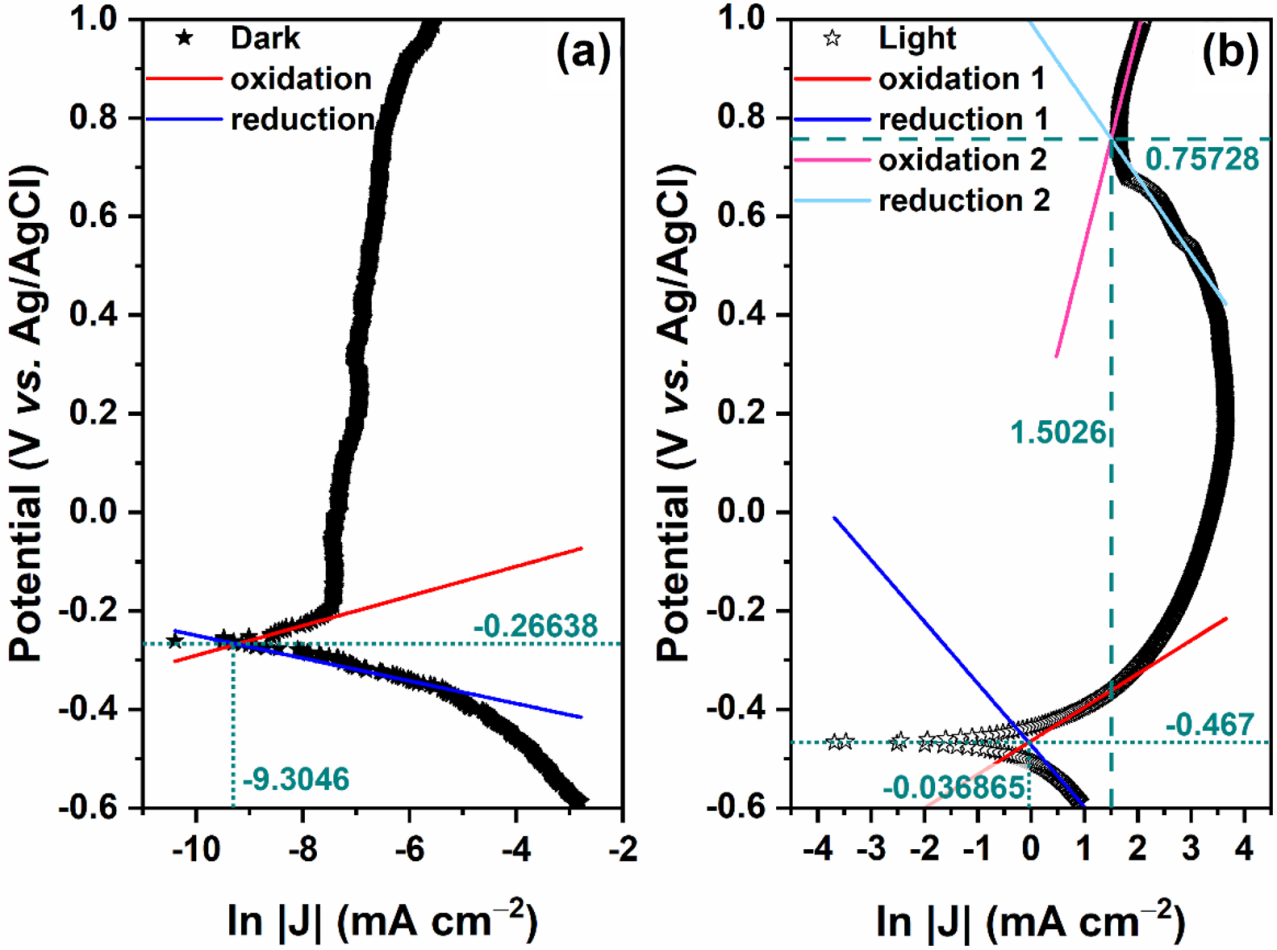
Tafel’s plot: (**a**) dark and (**b**) light.

We would like to point out that in the region of the visible spectrum, the photoelectrode showed a light harvesting efficiency (LHE) from 36.67 to 47.23% and separation charges (η_sep_) around 48%, at applied potentials from 0.5 to 0.8 V_RHE_. Traditionally, the thermodynamic potential required for the water decomposition reaction is nearly 1.23 V_RHE_. Because of the superpotentials at the cathode and anode, a voltage higher than 1.6 V is required for the electrolyzer to operate under a current density of around 1 A cm^–2^. In this case, almost 33.5–40% of electricity can be lost, resulting in a low overall energy conversion efficiency^50,51^. In practice, in order to minimize the energy loss, water electrolysis should be performed in highly acidic or alkaline conditions^52^. However, in this work, the data presented in Fig. 7 (representing the Tafel’s plot), suggest that the action of superoxide ions (O^2–^), resulting from the first redox step, can act in the second redox step (where a drop in photocurrent density occurs) in order to increase the potential energy necessary for the kinetic process of separation/transfer of photogenerated charges. Therefore, water splitting can be conducted in a sustainable manner in an electrochemical reaction.

The excess potential (η) was estimated by the intersection of the anodic and cathodic inclinations and, according to the Tafel’s kinetic-electrochemical model, is represented by the Eq. (3), where J is the current density, α represents the excess of anodic and cathodic potential, and β is the Tafel's slope of the anodic (or cathodic) reaction^53^.

3 $$\upeta =\upalpha \pm\upbeta \times \mathrm{ln}\left|\mathrm{J}\right|$$

This mathematical model is based on the kinetics and thermodynamics of the reactions involved on the surface of the photoelectrode, making possible to predict the potential and density of corrosion current by extrapolating the anodic and cathodic slopes^54^. In general, the higher the value of η the better the anti-corrosion properties. 54 From the Tafel’s plot shown in Fig. 7 it was possible to obtain some parameters, which are summarized in Table 2.

**Table 2:** Parameters obtained from the Tafel plots for the BVO photoelectrode.

Conditions	Parameters						
	β_anodic_	β_cathodic_	α_anodic_	α_cathodic_	$\upeta$ (V_RHE_)	J_corr_ (mA cm^–2^)	V_op_ (V_RHE_)
Dark	0.029	– 0.023	0.010	– 0.480	1.05	9.1001E–5	1.19
Light 1	0.068	– 0.125	–0.464	– 0.472	0.84	0.9638	1.24
Light 2	0.431	– 0.156	0.110	0.992	2.07	4.4933	1.67

In particular, the photoelectrode under illumination reached a value of η = 2.07 V_RHE_, well above the aforementioned minimum potential. In addition, values of the operational potential (V_op_) required for water splitting at a given current density (J) were obtained by the Eq. (4)^55^, and are also listed in Table 2.

4 $${\mathrm{V}}_{\mathrm{op}}=1.23+\left({\upbeta }_{\mathrm{anodic}}\times \mathrm{ln}\left|{\mathrm{J}}_{\mathrm{anodic}}\right|\right)+\left({\upbeta }_{\mathrm{cathodic}}\times \mathrm{ln}|{\mathrm{J}}_{\mathrm{cathodic}}|\right)+\left(\mathrm{J}\times \mathrm{A}\times {\mathrm{R}}_{\mathrm{p}}\right)$$

The A parameter is the electrode area and R_p_ represents the polarization resistance, which is obtained by the Eq. (5)^56^.

5 $${R}_{p}=\left|\frac{\left({\beta }_{anodic}\times {\beta }_{cathodic}\right)}{2.3\times {J}_{corr}\times \left({\beta }_{anodic}+{\beta }_{cathodic}\right)}\right|$$

Notice that the values of V_op_ and η are close each other, showing excellent agreement with the expected data from the Tafel’s plot towards the explored current densities. At the operating point, the efficiency for energy conversion ($\mathrm{PCE}={\mathrm{V}}_{\mathrm{op}}\times {\mathrm{J}}_{\mathrm{op}}/\mathrm{P}$) was estimated to be around 9.3%. Regarding the high density of photogenerated current (high HC–STH efficiency) we have reported, a reasonable approach was described in this manuscript, that is, when Bi_46_V_8_O_89_ comes into contact with the Bi_4_O_7_/Bi_3.33_(VO_4_)_2_O_2_ interface, an adjustable virtual *p–n–p* or *n–p–n* junction is created, with the rectifying contact acting afterward as a conventional *p–n* junction. For this reason, photogenerated charges can be efficiently separated by the ternary heterostructure, thus providing such a high density of photogenerated current. At the microscopic level, moreover, the following mechanisms may be also taken into consideration. It is well-known that the efficiency of the photogenerated *e*^*–*^*/h*^+^ pairs separation is a critical factor for the photoelectrochemical performance^39^. Because of the Bi^3+^ and Bi^5+^ coexistence in the present BVO material, there is a scenario with bridges for local charge transfer and to reinforce the photogenerated electrons flow. Furthermore, Bi^5+^ should act as a charge trap for electrons, thus delaying the recombination of electrons and photogenerated holes^39,57^. For the same reason (the ions coexistence), and with Bi_4_O_7_ acting as a photosensitizer, singlet oxygen is formed after excitation by a triplet–triplet energy transfer step. This this kind of electrophilic oxygen plays also an important role in increasing the efficiency in the photoelectrochemical oxidation process, leading to an improvement in the HC–STH efficiency for energy conversion^38,57^.

Further insights into the feature of electrical transport process across the photoelectrode can be theoretically also obtained via electrochemical impedance spectroscopy (EIS). The measurements were carried out with a 20 mV alternating-current (AC) potential, together with a direct-current (DC bias) potential at + 1.23 V_RHE_. The measured impedance data were processed using Nyquist plots (as observed in Figure S6), from which incidence of three semicircles was noted and simulated using an appropriate professional software (NOVA 2.1.3—Metrohm Autolab BV, Netherlands). The impedance data shown in Figure S6 have been fitted by the equivalent circuit represented in Figure S7, where a non-ideal Debye model involving three resistance-constant phase elements (Ri–Qi) networks, all connected in series, with each set of R and Q elements linked in parallel, plus a series resistance (Rs) coupled, has been considered, being Rs the resistance of the used solution (Na_2_SO_4_, 0.6 M). In practice, materials whose impedance data follow the non-ideal model show depressed semicircles below the real axis in Nyquist plots for (1–*n*)/2 angles. For *n* = 1, such a model reduces to the classical Debye’s scenario. In our work, we verified *n* values to be in the range of 0.69–0.96, approaching to unity for the high-frequency semicircle. Table 3 summarize the electrochemical parameters derived from Nyquist plots, according to the impedance measurements performed on the BVO photoelectrode.

**Table 3:** Electrochemical parameters derived from Nyquist plots, according to the impedance measurements performed on the BVO photoelectrode.

Condition	Electrochemical parameters							
	Rs (Ω)	R_1_ (Ω)	C_1_ (μF)	R_2_ (Ω)	C_2_ (μF)	R_3_ (Ω)	C_3_ (μF)	${\tau}_{2}$(ms)
Dark	0.0	4,831	14.22	77.1	0.009	3,147.7	55.8	0.0007
Light	0.0	13.73	1.92	1916.4	25.72	21.2	0.03	49.3

The equivalent values we finally found for capacitance fall in the range of the expected values for the different contributions we have identified, in line with similar results reported in the literature (Ref. 59 cited). In particular, the R_1_–Q_1_ network, attributed to the polycrystalline photoelectrode, with C_1_ values in the order of 10^–8^ F, should arise from the grain-boundary contribution. Observing the electrical response arising from the corresponding bulk would likely need to expand the measurements to higher frequencies (i.e., above 100 kHz). The values of Q_2_ and Q_3_ are also compatible in magnitude order (significantly higher than expected for grain-boundary or bulk effects) with the designated interface and diffusion contributions. The constant phase element in parallel with the resistors (R_1_, R_2_ and R_3_) can be converted into the capacitances (C_1_, C_2_ and C_3_) through the Eq. (6), 58 where C is the resulting capacitance, Y_0_ is the admittance value of the constant phase element, R is the resistance value and *n* is the exponent of the constant phase element^58^.

6 $$\mathrm{C}= {\mathrm{Y}}_{0}^{1/n}\times {\mathrm{R}}^{(1/n-1)}$$

As expected for charge transfer (R_1_) and diffusion (R_3_) processes, results shown in Table 3 indicate that presence of light is responsible for an increase in conductivity. Also observed in this table, R_2_ is quite high under illumination, demonstrating that there is less recombination of electrons and, thus, enhanced chemical capacitance effect (C_2_) at the BVO/electrolyte interface. The lifetime of the photoinjected electrons (${\tau}_{2}$) can be and was estimated using the simple expression represented by the Eq. (7). 59

7 $${\tau}_{2}={\mathrm{R}}_{2}\times {\mathrm{C}}_{2}$$

The combination of these two electrical parameters produces a very high value of lifetime ${\tau}_{2}$ (also presented in Table 3) for the photoinjected electrons, accounting for the high values we in fact also observed for the HC–STH efficiency (Figure S5). Furthermore, the excellent photoelectrochemical performance is the result of the exceptional electrical conductivity level of the ITO and the used active area of the photoelectrode ~ 0.2 cm^2^^60^.

Finally, the onset potential of the BVO film was also determined by processing the photocurrent data in a J^2^ versus applied potential plot (as shown in Figure S8), at which point the potential where the current starts to increase, that is, a reaction begins to occur, is identified. The obtained value was 0.33 V_RHE_, much lower than the values found in bismuth- and vanadium-based films such as Bi_46_V_8_O_89_ (0.53 V_RHE_), BiVO_4_ (0.50 V_RHE_), BiVO_4_/Bi_4_V_2_O_11_ (0.54 V_RHE_) and W/BiVO_4_/Bi_4_V_2_O_11_ (0.66 V_RHE_)^11^. This result confirms that the ternary heterojunction formed in our BVO film improved the charge-transfer kinetics through the photoelectrode/electrolyte interface, that is, without the use of dopants or cocatalysts.

## Discussion and concluding remarks

**Figure 8:**
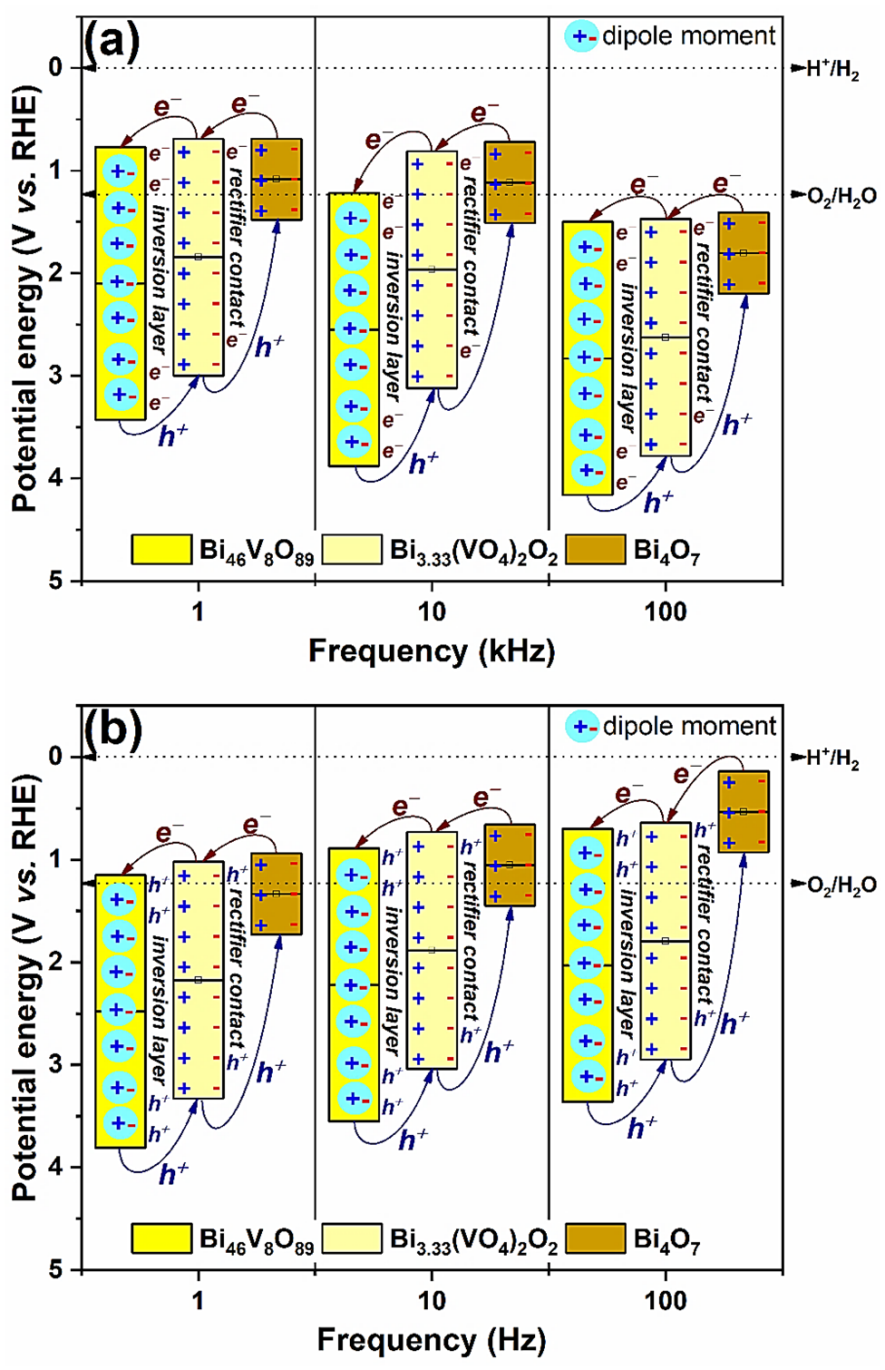
Schematic diagram of Bi_4_O_7_/Bi_3.33_(VO_4_)_2_O_2_/Bi_46_V_8_O_89_ heterojunction with a rectifier contact and a polarization-induced inversion layer. (**a**) Ternary heterojunction *p–p–p* (virtual *n–p–n*), and (**b**) ternary heterojunction *n–n–n* (virtual *p–n–p*).

Due to the intimate contact between Bi_46_V_8_O_89_ and Bi_3.33_(VO_4_)_2_O_2_ nanoplates, a sufficiently high internal polar electric field is created between both phases, resulting in the formation of a minority electron inversion layer (Fig. 8a) or minority holes (Fig. 8b) at the interface, as verified from the Mott–Schottky data (Fig. 5). The difference between the effective working functions of both semiconductors is the driving force required to achieve the electronic equilibrium through a transfer of charges between them and the creation of a spatial-charge region at the heterojunction interface. Then, charges of the same polarity are repelled from the interface, while those of the opposite polarity are attracted to it. Accordingly, when the ionic-conductor Bi_46_V_8_O_89_ (with an inherent-like ability to form dipoles) is in contact with Bi_3.33_(VO_4_)_2_O_2_, most of the electrons will be depleted from the interface between them, while the minority electrons (or minority holes) will be attracted to this interface, thereby reversing the nature (type) of conductivity. This is in agreement with the electrochemical behavior exhibited by the Mott–Schottky data (Fig. 5), which revealed typical characteristics of *n*-type as well as *p*-type semiconductors.

With respect to the Bi_4_O_7_/Bi_3.33_(VO_4_)_2_O_2_ interface, formation of a rectifier contact occurs, where the energy difference causes the transfer of electrons from Bi_4_O_7_ to Bi_3.33_(VO_4_)_2_O_2_ in order to match the Fermi levels under open circuit conditions. Fixed charges appear at the junction interface on both sides. In Bi_4_O_7_, a space charge region is formed due to the positively ionized donor atoms (Figs. 8a, b). In Bi_3.33_(VO_4_)_2_O_2_ there is, therefore, an accumulation of minority electrons (Fig. 8a) or minority holes (Fig. 8b) at the interface. For this junction, the electric field corresponding to the contact potential difference is directed from Bi_4_O_7_ to Bi_3.33_(VO_4_)_2_O_2_, and its decrease, because of direct polarization, means a decrease in the potential barrier height for the electrons in Bi_4_O_7_; this scenario allows an easier motion of the electrons from Bi_4_O_7_ to Bi_3.33_(VO_4_)_2_O_2_ and, therefore, an increase in the photogenerated current. This behavior is similar to that from a Schottky diode^61,62^.

Furthermore, as previously discussed, the easy electron injection of CB from Bi_4_O_7_ to CB from Bi_3.33_(VO_4_)_2_O_2_, and later from CB from Bi_3.33_(VO_4_)_2_O_2_ to CB from Bi_46_V_8_O_89_ in the heterostructure, is favored because the CB and VB bands of Bi_4_O_7_ are more negative than the corresponding bands of Bi_3.33_(VO_4_)_2_O_2_, and the CB and VB bands of Bi_3.33_(VO_4_)_2_O_2_ are more negative than the corresponding bands of Bi_46_V_8_O_89_.

In summary, due to a self-polarization-like event of the Bi_46_V_8_O_89_ ionic-conductor, and depending on the applied potential and considered frequency of operation, the nature of the conductivity across this designed ternary Bi_4_O_7_/Bi_3.33_(VO_4_)_2_O_2_/Bi_46_V_8_O_89_ heterojunction can be modified, becoming either a virtually *p–n–p* type heterojunction at low frequencies: 1–100 Hz (Fig. 8b), or a *n–p–n* type heterojunction at high frequencies: 1–100 kHz (Fig. 8a). These findings are exclusive, as they open a new design strategy to be considered for construction of multifunctional, high-efficiency hybrid photoelectrodes for sustainable artificial photosynthesis.

## Experimental section

### Preparation of the precursor solution and deposition on the transparent conductive ITO-coated glass

All raw materials were used as received, without further purification. Ten millimoles of Bi_5_O(OH)_9_(NO_3_)_4_ was dissolved in 50 mL of CH_3_COOH (99.7%) to get the solution “A”. In another recipient, 10 mmol of NH_4_VO_3_ (99%) was dissolved in 50 mL of NH_4_OH (28%), producing the solution “B”. Then, the solutions A and B were mixed under stirring at 80 °C and 50 mmol of citric acid was added to the mixture. The obtained gel was oven-dried at 150 °C for 24 h. The precursor solution was produced by diluting the sintered powder in C_3_H_8_O_7_ (99.5%), followed by deposition on the transparent conducting ITO coated glass by applying the spin-coating method. The deposited bismuth- and vanadium-containing film, abbreviated as BVO, underwent the crystallization process in a muffle at 5 °C min^–1^ (heating rate) from room temperature to 450 °C, plus annealing for 5 h.

### Characterization

The morphology of the BVO film was investigated by scanning electron microscopy (SEM, microscope model VEGA3 TESCAN) operated at 5.0 kV, coupled to an energy dispersive spectroscopy (EDS) mapping device (Oxford Instruments, Model 51–ADD0007) with 20 kV accelerating voltage. The crystalline phases of the films were determined using an X-ray diffractometer (XRD 6,000, Shimadzu). The data were collected from 10 to 80° at a step-width of 0.2°, scan-speed of 2.0° min^–1^, sampling pitch of 0.02° and preset time of 5 s, at 40 kV, 30 mA, using CuKα radiation (λ = 1.540560 Å). Silicon was used as an external standard sample. Structure refinement through Rietveld was performed using the GSAS + EXPGUI program package^63,64^. Further information on the structural phases involved was extracted from Raman spectroscopy. The measurements were performed towards the 40 to 1,500 cm^–1^ region, using a Horiba LabRAM HR Evolution spectrometer, with a 532 nm laser line. The system was calibrated with a 520.7 cm^–1^ silicon wafer, and the LabSpec 6 software was used for data collection within a spectral resolution of 1 cm^–1^. The diffuse reflectance spectra (DRS) were collected within a resolution of 0.1 nm by using a UV–Vis spectrophotometer (UV–2501 PC Shimadzu), equipped with an ISR 240A integration sphere (and UVPC program), while BaSO_4_ was used as reference material (100% transmission). The direct band-gap energies were calculated via the following Tauc equation:

8 $$(\upalpha {\text{h}}\upnu )^{{2}} \, = \,{\text{A}}({\text{h}}\upnu \, - \,{\text{E}}_{{\text{g}}} )$$

where A is a constant, hν the light energy, E_g_ the optical band-gap energy and α refers to the absorption coefficient. The fraction of transmitted light was measured as reflectance, and the Kubelka–Munk radiative transfer model was employed to extract the α parameter. This model is described by the equation below:

9 $$\mathrm{F}\left(\mathrm{R}\right)= \frac{{\left(1-\mathrm{R}\right)}^{2}}{2{\mathrm{R}}}=\frac{{\upalpha }}{{\mathrm{S}}}$$

where F(R) is the Kubelka–Munk function, S the scattering coefficient and R stands for the absolute reflectance. If the scattering coefficient is wavelength independent, then F(R) is proportional to α and the Tauc’s plots can be made using F(R) instead of α^65,66^.

### Photoelectrochemical measurements and electrochemical impedance spectroscopy

The photoelectrochemical measurements were carried out using a potentiostat AUTOLAB Potentiostat–Galvanostat PGSTAT204. The system uses a standard three-electrode cell with an Ag/AgCl (3.0 M KCl) reference electrode, a platinum metalized FTO substrate as a counter electrode, a working electrode with irradiation area of 0.2 cm^2^, and a scan-rate of 20 mV s^–1^. A Na_2_SO_4_ aqueous solution (with 0.5 M and pH = 6.6) was used as electrolyte. The prepared BVO film, tested as the working electrode, was connected to a copper tape in order to measure the photoactivity. The current-potential curves were recorded in the dark and under back illumination. For converting the obtained potential *vs.* Ag/AgCl to RHE (Reversible Hydrogen Electrode), the following expression was used:

10 $${\mathrm{V}}_{\mathrm{RHE}}={\mathrm{V}}_{\mathrm{Ag}/\mathrm{Ag}\mathrm{Cl}}+0.059\mathrm{pH}+{\mathrm{V}}_{\mathrm{Ag}/\mathrm{AgCl}}^{\mathrm{o}}$$

The $${\mathrm{V}}_{\mathrm{Ag/AgCl}}^{\mathrm{o}} \left(\mathrm{KCl}\, 3\, \mathrm{M}\right)= 0.197\,\mathrm{at}\,25\,^{\circ}{\rm C} $$.

When the BVO photoelectrode behaved like a photoanode, the applied-bias-compensated half-cell solar-to-hydrogen (HC–STH) efficiency was calculated from the following equation:

11 $$\mathrm{HC}-\mathrm{STH}=\frac{\left|{\mathrm{J}}_{\mathrm{light}}-{\mathrm{J}}_{\mathrm{dark}}\right|\times \left({\mathrm{V}}_{{\mathrm{O}}_{2}/{\mathrm{H}}_{2}\mathrm{O}}-{\mathrm{V}}_{\mathrm{RHE}}\right)\times\upeta }{{\mathrm{P}}_{\mathrm{light}}}$$

On the other hand, when the BVO photoelectrode behaved like a photocathode, the equation below was used:

12 $$\mathrm{HC}-\mathrm{STH}=\frac{\left|{\mathrm{J}}_{\mathrm{light}}-{\mathrm{J}}_{\mathrm{dark}}\right|\times \left({\mathrm{V}}_{\mathrm{RHE}}-{\mathrm{V}}_{{\mathrm{H}}^{+}/{\mathrm{H}}_{2}}\right)\times\upeta }{{\mathrm{P}}_{\mathrm{light}}}$$

where V_RHE_ is the applied potential, J_dark_ and J_light_ are the photocurrent densities in the dark and under irradiated light, respectively, P_light_ is the power density of the irradiated light, while V_O2/H2O_ and V_H_^+^_/H2_ represent the equilibrium potentials of oxygen (+ 1.23 V_RHE_) and hydrogen (0 V_RHE_) evolution, respectively. Finally, the faraday efficiency is symbolized by η^67–69^.

The electrochemical impedance spectroscopy was performed using the AUTOLAB Potentiostat–Galvanostat PGSTAT204 equipped with the FRA32M module. The standard three-electrode cell configuration was also used, together with the Na_2_SO_4_ aqueous solution (0.5 M) as electrolyte. The impedance measurements were carried out under a + 1.23 V_RHE_ DC potential bias, with an amplitude of 0.01 V_RMS_, where RMS is the *root mean square*, in the frequency range of 100 mHz to 100 kHz. An Xe Lamp (λ > 450 nm, 100 mW cm^–2^) was used as the light source. The measured data were processed using Nyquist plots and fitted using the NOVA 2.1.3 software (Metrohm Autolab BV, Netherlands).

It was used as a source of light a 300 W Xenon arc lamp (Newport Ozone Free Model 6,258). The equipment used to measure the intensity (power density) of the irradiated light was a Newport model 1916-R optical power meter equipped with an UV sensor (Newport UV 818-UV/DB). An AM 1.5 filter in front of the light output was also used as the sun simulator.

### Mott–Schottky data acquisition

The spectra were collected using also the AUTOLAB Potentiostat–Galvanostat PGSTAT204 equipped with the FRA32M module, and a cell configuration with three electrodes: a reference electrode of Ag/AgCl (3.0 M KCl), a platinum metalized FTO substrate as counter electrode wire and a working electrode (the BVO film) with 0.2 cm^2^ area in the dark, applying a potential from –0.7 to + 0.7 V_Ag/AgCl_ in the frequency range of 1 Hz to 100 kHz, using a step of 0.03 V_Ag/AgCl_ and amplitude of 0.01 V_RMS_. The measured spectra were fitted using the NOVA 2.1.3 software (Metrohm Autolab BV, Netherlands). Again, the Na_2_SO_4_ solution (0.5 M) was used as electrolyte for these electrochemical measurements.

## Supplementary information

Supplementary information

## References

[1] Duan L, Tong L, Xu Y, Sun L (2011). Visible light-driven water oxidation—from molecular catalysts to photoelectrochemical cells. Energy Environ. Sci..

[2] Gratzel M (2001). Photoelectrochemical cells. Nature.

[3] Liu Q (2015). A multijunction of ZnIn_2_S_4_ nanosheet/TiO_2_ film/Si nanowire for significant performance enhancement of water splitting. Nano Res..

[4] Swierk JR, Mallouk TE (2013). Design and development of photoanodes for water-splitting dye-sensitized photoelectrochemical cells. Chem. Soc. Rev..

[5] Walter MG (2010). Solar water splitting cells. Chem. Rev..

[6] Li J, Wu N (2015). Semiconductor-based photocatalysts and photoelectrochemical cells for solar fuel generation: a review. Catal. Sci. Technol..

[7] Li Z, Luo W, Zhang M, Feng J, Zou Z (2013). Photoelectrochemical cells for solar hydrogen production: current state of promising photoelectrodes, methods to improve their properties, and outlook. Energy Environ. Sci..

[8] Kim TL, Choi M-J, Jang HW (2018). Boosting interfacial charge transfer for efficient water-splitting photoelectrodes: progress in bismuth vanadate photoanodes using various strategies. MRS Commun..

[9] Peerakiatkhajohn P, Yun J-H, Wang S, Wang L (2016). Review of recent progress in unassisted photoelectrochemical water splitting: from material modification to configuration design. J. Photon. Energy.

[10] dos Santos WS (2016). Photoelectrochemical water oxidation over fibrous and sponge-like BiVO_4_/β-Bi_4_V_2_O_11_ photoanodes fabricated by spray pyrolysis. Appl. Catal. B Environ..

[11] dos Santos WS (2016). A hole inversion layer at the BiVO_4_/Bi_4_V_2_O_11_ interface produces a high tunable photovoltage for water splitting. Sci. Rep..

[12] dos Santos WS (2018). Bismuth vanadate photoelectrodes with high photovoltage as photoanode and photocathode in photoelectrochemical cells for water splitting. Chemsuschem.

[13] Lopes OF, Carvalho KTG, Avansi W, Ribeiro C (2017). Growth of BiVO_4_ nanoparticles on a Bi_2_O_3_ surface: effect of heterojunction formation on visible irradiation-driven catalytic performance. J. Phys. Chem. C.

[14] Lv C, Chen G, Sun J, Zhou Y (2016). Construction of α–β phase junction on Bi_4_V_2_O_11_ via electrospinning retardation effect and its promoted photocatalytic performance. Inorg. Chem..

[15] Liu Z, Niu J, Feng P, Sui Y, Zhu Y (2014). One-pot synthesis of Bi_24_O_31_Br _1_0/B_i_4_V_2_O1_1 heterostructures and their photocatalytic properties. RSC Adv..

[16] Lv T (2017). Facile synthesis of CdS/Bi_4_V_2_O_11_ photocatalysts with enhanced visible-light photocatalytic activity for degradation of organic pollutants in water. Dalton Trans..

[17] Liu T, Mao YG, Peng Y (2018). Synthesis of Bi_2_O_3_–Bi_4_V_2_O_11_ heterojunctions with high interface quality for enhanced visible light photocatalysis in degradation of high-concentration phenol and MO dyes. CrystEngComm.

[18] Cong Y (2016). Enhanced Photoelectrocatalytic Activity Of A Novel Bi_2_O_3_–BiPO_4_ composite electrode for the degradation of refractory pollutants under visible light irradiation. Ind. Eng. Chem. Res..

[19] Kalanur SS, Yoo I-H, Park J, Seo H (2017). Insights into the electronic bands of WO_3_/BiVO_4_/TiO_2_, revealing high solar water splitting efficiency. J. Mater. Chem. A.

[20] Zhu M (2017). Boosting the visible-light photoactivity of BiOCl/BiVO_4_/N-GQD ternary heterojunctions based on internal Z-scheme charge transfer of N-GQDs: simultaneous band gap narrowing and carrier lifetime prolonging. ACS Appl. Mater. Interfaces.

[21] Hwang SW (2019). Solution-processed TiO_2_/BiVO_4_/SnO_2_ triple-layer photoanode with enhanced photoelectrochemical performance. J. Alloys Compd..

[22] McKone JR, Lewis NS, Gray HB (2014). Will solar-driven water-splitting devices see the light of day?. Chem. Mater..

[23] Prévot MS, Sivula K (2013). Photoelectrochemical tandem cells for solar water splitting. J. Phys. Chem. C.

[24] Zhang K, Ma M, Li P, Wang DH, Park JH (2016). Water splitting progress in tandem devices: moving photolysis beyond electrolysis. Adv. Energy Mater..

[25] Grätzel M (1980). Photochemical methods for the conversion of light into chemical energy. Ber. Bunsen-Ges. Phys. Chem..

[26] Darriet J, Launay JC, Zúniga FJ (2005). Crystal structures of the ionic conductors Bi_46_M_8_O_89_ (M = P, V) related to the fluorite-type structure. J. Solid State Chem..

[27] Kuang X, Payne JL, Farrell JD, Johnson MR, Evans IR (2012). Polymorphism and oxide ion migration pathways in fluorite-type bismuth vanadate, Bi_46_V_8_O_89_. Chem. Mater..

[28] Kumada N (2011). Preparation and crystal structure of a new bismuth vanadate, Bi_3.33_(VO_4_)_2_O_2_. Mater. Res. Bull..

[29] Guan H, Feng Y (2015). Facile synthesis and purplish blue luminescence of the binary mixed valence compound Bi_4_O_7_ microcrystals. Mater. Lett..

[30] Sun M (2017). Fabrication of a novel Z-scheme g-C_3_N_4_/Bi_4_O_7_ heterojunction photocatalyst with enhanced visible light-driven activity toward organic pollutants. J. Colloid Interface Sci..

[31] Dinnebier RE, Ibberson RM, Ehrenberg H, Jansen M (2002). The crystal structures of the binary mixed valence compound Bi_3_^(III)^Bi^(V)^O_7_ and isotypic Bi_3_SbO_7_ as determined by high resolution X-ray and neutron powder diffraction. J. Solid State Chem..

[32] Buixaderas E (2015). Compositional behavior of Raman-active phonons in Pb(Zr_1−x_Ti_x_)O_3_ ceramics. Phys. Rev. B.

[33] Đorđević T, Karanović L (2014). A new anion-deficient fluorite-related superstructure of Bi_28_V_8_O_62_. J. Solid State Chem..

[34] Zhang G, Cai L, Zhang Y, Wei Y (2018). Bi^5+^, Bi^(3–x)+^, and oxygen vacancy induced BiOCl_x_I_1−x_ solid solution toward promoting visible-light driven photocatalytic activity. Chem. Eur. J..

[35] Hardcastle FD, Wachs IE, Eckert H, Jefferson DA (1991). Vanadium(V) environments in bismuth vanadates: a structural investigation using Raman spectroscopy and solid state ^51^V NMR. J. Solid State Chem..

[36] Hardcastle FD, Wachs IE (1991). Determination of vanadium–oxygen bond distances and bond orders by Raman spectroscopy. J. Phys. Chem..

[37] Shvets P, Dikaya O, Maksimova K, Goikhman A (2019). A review of Raman spectroscopy of vanadium oxides. J. Raman Spectrosc..

[38] Hu Y (2016). Temperature-induced phase changes in bismuth oxides and efficient photodegradation of phenol and *p*-chlorophenol. J. Hazard. Mater..

[39] Jia Y (2020). Oxygen vacancy rich Bi_2_O_4_–Bi_4_O_7_–BiO_2-x_ composites for UV–vis–NIR activated high efficient photocatalytic degradation of bisphenol A. J. Hazard. Mater..

[40] Watanabe A (2001). Preparation and characterization of a new triclinic compound Bi_3.5_V_1.2_O_8.25_ to show the known phase Bi_4_V_2_O_11_ to be nonexistent as a single phase. J. Solid State Chem..

[41] Awasthi RR, Das B (2019). Structural transition and tunable optical, morphological and magnetic properties of Mn-doped BiFeO_3_ films. Optik.

[42] Thanikaikarasan S, Karthickprabhu S, Dhanasekaran D, Vijayan V (2019). Physical, chemical and optical properties of CdSe and CdSe: Zn thin films obtained through low cost electrochemical route. Mater. Today: Proc..

[43] Ganguly P, Hegde MS (1988). Evidence for double valence fluctuation in metallic oxides of lead. Phys. Rev. B.

[44] Sajjad S, Leghari SAK, Zhang J (2013). Nonstoichiometric Bi_2_O_3_: efficient visible light photocatalyst. RSC Adv..

[45] Rakesh K (2011). Role of doping-induced photochemical and microstructural properties in the photocatalytic activity of InVO_4_ for splitting of water. J. Mater. Sci..

[46] Gelderman K, Lee L, Donne SW (2007). Flat-band potential of a semiconductor: using the Mott–Schottky equation. J. Chem. Educ..

[47] Lee KJ, Elgrishi N, Kandemir B, Dempsey JL (2017). Electrochemical and spectroscopic methods for evaluating molecular electrocatalysts. Nat. Rev. Chem..

[48] Xie Y, Ju Y, Toku Y, Morita Y (2017). Fabrication of Fe_2_O_3_ nanowire arrays based on oxidation-assisted stress-induced atomic-diffusion and their photovoltaic properties for solar water splitting. RSC Adv..

[49] Oliveira AT (2018). High water oxidation performance of W-doped BiVO_4_ photoanodes coupled to V_2_O_5_ rods as a photoabsorber and hole carrier. Sol. RRL.

[50] Xu W, Scott K (2010). The effects of ionomer content on PEM water electrolyser membrane electrode assembly performance. Int. J. Hydrog. Energy.

[51] Zhou H (2018). Water splitting by electrolysis at high current densities under 1.6 volts. Energy Environ. Sci..

[52] Anantharaj S (2018). Precision and correctness in the evaluation of electrocatalytic water splitting: revisiting activity parameters with a critical assessment. Energy Environ. Sci..

[53] Li J, Guo L, Zhou J, Song Q, Liang Z (2018). Enhancing the photoelectrochemical performance of BiVO_4_ by decorating only its (040) facet with self-assembled Ag@AgCl QDs. J. Solid State Electrochem..

[54] Kakaei K, Esrafili MD, Ehsani A, Kakaei K, Esrafili MD, Ehsani A (2019). Interface Science and Technology.

[55] Surendranath Y, Bediako DK, Nocera DG (2012). Interplay of oxygen-evolution kinetics and photovoltaic power curves on the construction of artificial leaves. PNAS USA.

[56] Holze R, Elektrochemie CH, Hamann W (1999). Vielstich Wiley-VCH Verlagsgesellschaft mbH, Weinheim 1998, 602 S., 301 Abb., 35 Tab., brosch., DM 98, ISBN 3-527-27894-X. Chem. Ing. Tech..

[57] Zhong S, Xi Y, Chen Q, Chen J, Bai S (2020). Bridge engineering in photocatalysis and photoelectrocatalysis. Nanoscale.

[58] Orazem, M. E. & Tribollet, B. Time‐Constant Dispersion*. In: Electrochemical Impedance Spectroscopy* (ed John Wiley & Sons) Ch. 13, 233–263 (2008).

[59] Kundu A, Shit A, Nandi S (2017). Carbon dot assisted synthesis of nanostructured polyaniline for dye sensitized solar cells. Energy Fuels.

[60] Lee YW (2018). Unbiased biocatalytic solar-to-chemical conversion by FeOOH/BiVO_4_/perovskite tandem structure. Nat. Commun..

[61] Léonard F, Tersoff J (2000). Role of fermi-level pinning in nanotube schottky diodes. Phys. Rev. Lett..

[62] Lin F, Boettcher SW (2013). Adaptive semiconductor/electrocatalyst junctions in water-splitting photoanodes. Nat. Mater..

[63] Larson, A. C. & Dreele, R. B. V. General structural analysis system (GSAS). *Los Alamos National Laboratory Report LAUR*, 86–748 (2004).

[64] Toby B (2001). EXPGUI, a graphical user interface for GSAS. J. Appl. Crystallogr..

[65] Barton DG, Shtein M, Wilson RD, Soled SL, Iglesia E (1999). Structure and electronic properties of solid acids based on tungsten oxide nanostructures. J. Phys. Chem. B.

[66] Finlayson AP, Tsaneva VN, Lyons L, Clark M, Glowacki BA (2006). Evaluation of Bi–W-oxides for visible light photocatalysis. Phys. Status Solidi A.

[67] Dotan H, Mathews N, Hisatomi T, Grätzel M, Rothschild A (2014). On the solar to hydrogen conversion efficiency of photoelectrodes for water splitting. J. Phys. Chem. Lett..

[68] Hisatomi T, Kubota J, Domen K (2014). Recent advances in semiconductors for photocatalytic and photoelectrochemical water splitting. Chem. Soc. Rev..

[69] Pei L, Xu Z, Yan S, Zou Z (2017). Temperature-controlled evolution of microstructures that promote charge separation in a TaON photoanode for enhanced solar energy conversion. J. Mater. Chem. A.

