# Supplementary material for: Innovative multifunctional hybrid photoelectrode design based on a ternary heterojunction with super-enhanced efficiency for artificial photosynthesis
Source: Sci Rep. 2020 Jun 30;10:10669. doi: 10.1038/s41598-020-67768-y (PMC7327001; doi:10.1038/s41598-020-67768-y)
Supplement: Supplementary file 1 — Supplementary information [file 41598_2020_67768_MOESM1_ESM.docx]

Supplementary Information

**Innovative multifunctional hybrid photoelectrode design based on a ternary heterojunction with super–enhanced efficiency for artificial photosynthesis**

Wayler S. dos Santos^1^, Éder J. Carmo^1^, Yanela Mendez–González^2^, Lucas L. Nascimento^3^, Antônio O. T. Patrocínio^3^, Ruyan Guo^4^, Amar S. Bhalla^4^, Jean–Claude M’Peko^5^ and José D. S. Guerra^1,*^

^1^Group of Ferroelectrics and Multifunctional Materials, Institute of Physics, Federal University of Uberlandia, Uberlandia 38408–100, Minas Gerais, Brazil

^2^Physics Faculty/IMRE, University of Havana, 10400, Havana, Cuba

^3^Laboratory of Photochemistry and Materials Science, Institute of Chemistry, Federal University of Uberlandia, Uberlandia 38408–100, Minas Gerais, Brazil

^4^Multifunctional Electronic Materials and Devices Research Lab., Department of Electrical and Computer Engineering, College of Engineering, The University of Texas at San Antonio, San Antonio 78249, TX, USA

^5^São Carlos Institute of Physics, University of São Paulo, São Carlos 13560–970, São Paulo, Brazil

**Corresponding Author**

*Tel.: +55 34 32915924; Fax: +55 34 32394106; E–mail address: santos@ufu.br (J.D.S. Guerra).

Supplementary tables

**Table S1.** Crystallographic data for the BVO photoelectrode prepared in this work, as extracted from the XRD pattern simulation using the Rietveld method (χ^2^ = 1.46; R(F^2^) = 0.08; Rp = 0.05; Rwp = 0.07).

| **Data** | **Phase** | | |
| --- | --- | --- | --- |
|  | Bi_46_V_8_O_89_ | Bi_3.33_(VO_4_)_2_O_2_ | Bi_4_O_7_ |
| Space Group | P 1 21/c 1 | P –1 | P –1 |
| Crystal System | monoclinic | triclinic | triclinic |
| *a* (Å) | 19.734936 | 7.589915 | 6.762521 |
| *b* (Å) | 11.810645 | 8.118070 | 6.915585 |
| *c* (Å) | 20.021515 | 9.578054 | 7.918499 |
| α (^o^) | 90.0000 | 104.8396 | 71.3789 |
| β (^o^) | 106.6108 | 97.4860 | 88.0424 |
| γ (^o^) | 90.0000 | 112.4903 | 78.0835 |
| Density (g cm^–3^) | 8.499 | 6.147 | 9.174 |
| Volume (Å^3^) | 4471.916 | 509.581 | 343.172 |

**Table S2.** Wavenumber and the assigned vibrational modes for the BVO film.

| Peak | Raman data | |
| --- | --- | --- |
|  | Wavenumber (cm^–1^) | Vibrational modes |
| 1 | 75.61 | Bi–O (OBi_3_) |
| 2 | 94.03 | Bi–O (OBi_3_) |
| 3 | 133.63 | Bi–O (OBi_3_) |
| 4 | 186.31 | Bi–O (OBi_3_) |
| 5 | 298.12 | Bi–O (OBi_3_) |
| 6 | 347.02 | VO_4_ (symmetric ν_2_) |
| 7 | 467.43 | VO_4_ (symmetric ν_4_) |
| 8 | 498.08 | Bi–O (OBi_4_) |
| 9 | 594.51 | Bi–O (OBi_4_) |
| 10 | 640.64 | V–O–V |
| 11 | 698.17 | VO_4_ (anti–symmetric ν_3_) |
| 12 | 810.96 | V–O (symmetric ν_1_) |
| 13 | 813.74 | V–O (symmetric ν_1_) |
| 14 | 829.83 | V–O (symmetric ν_1_) |
| 15 | 994.67 | V^4+^=O |
| 16 | 997.29 | V^4+^=O |
| 17 | 1087.47 | V^5+^=O |

Supplementary figures


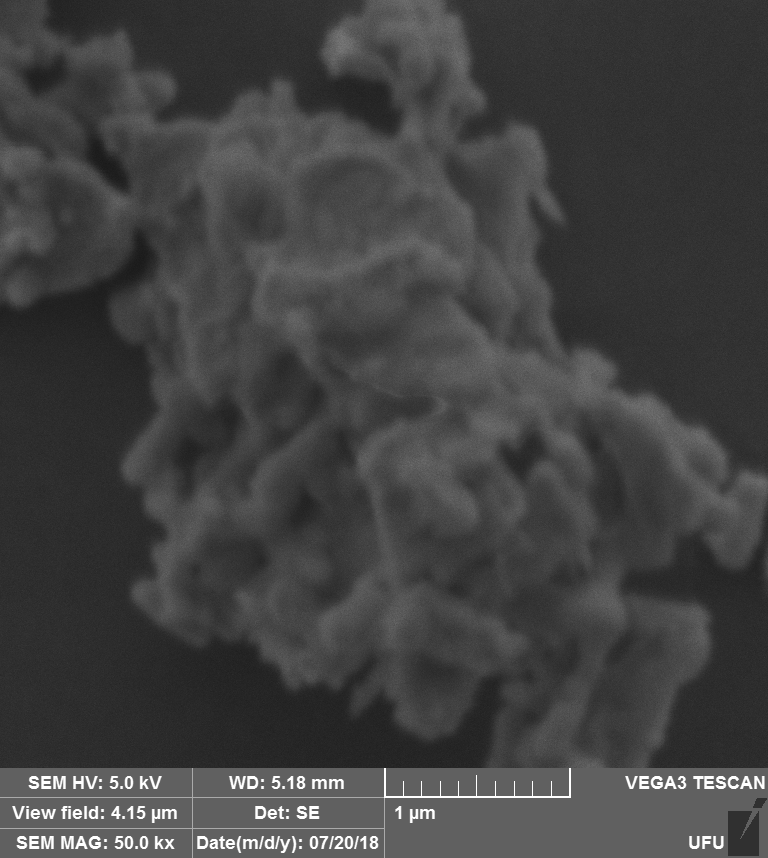


**Figure S1.** SEM image of the BVO film.


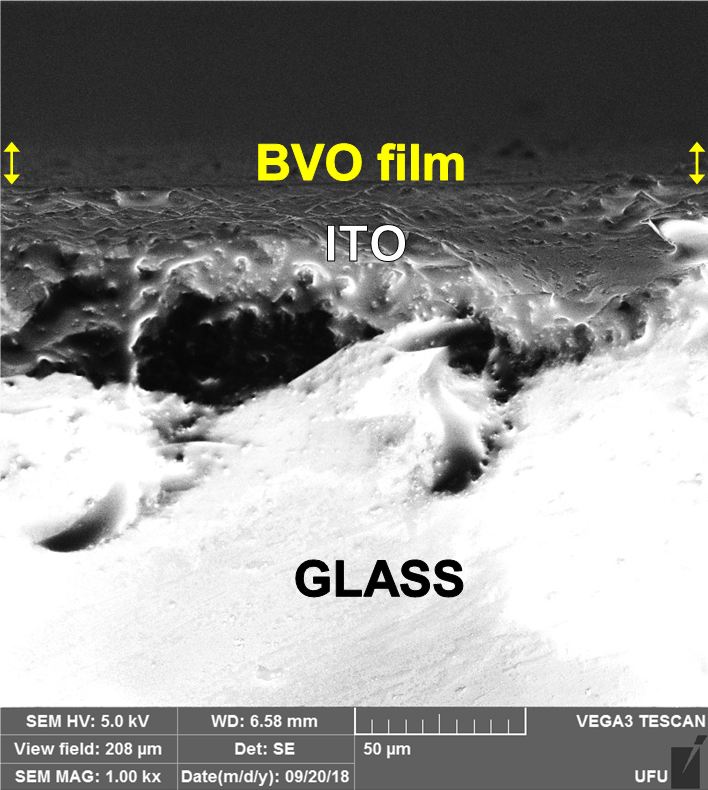


**Figure S2.** Cross–sectional SEM image of the BVO sample, showing the film thickness.

.


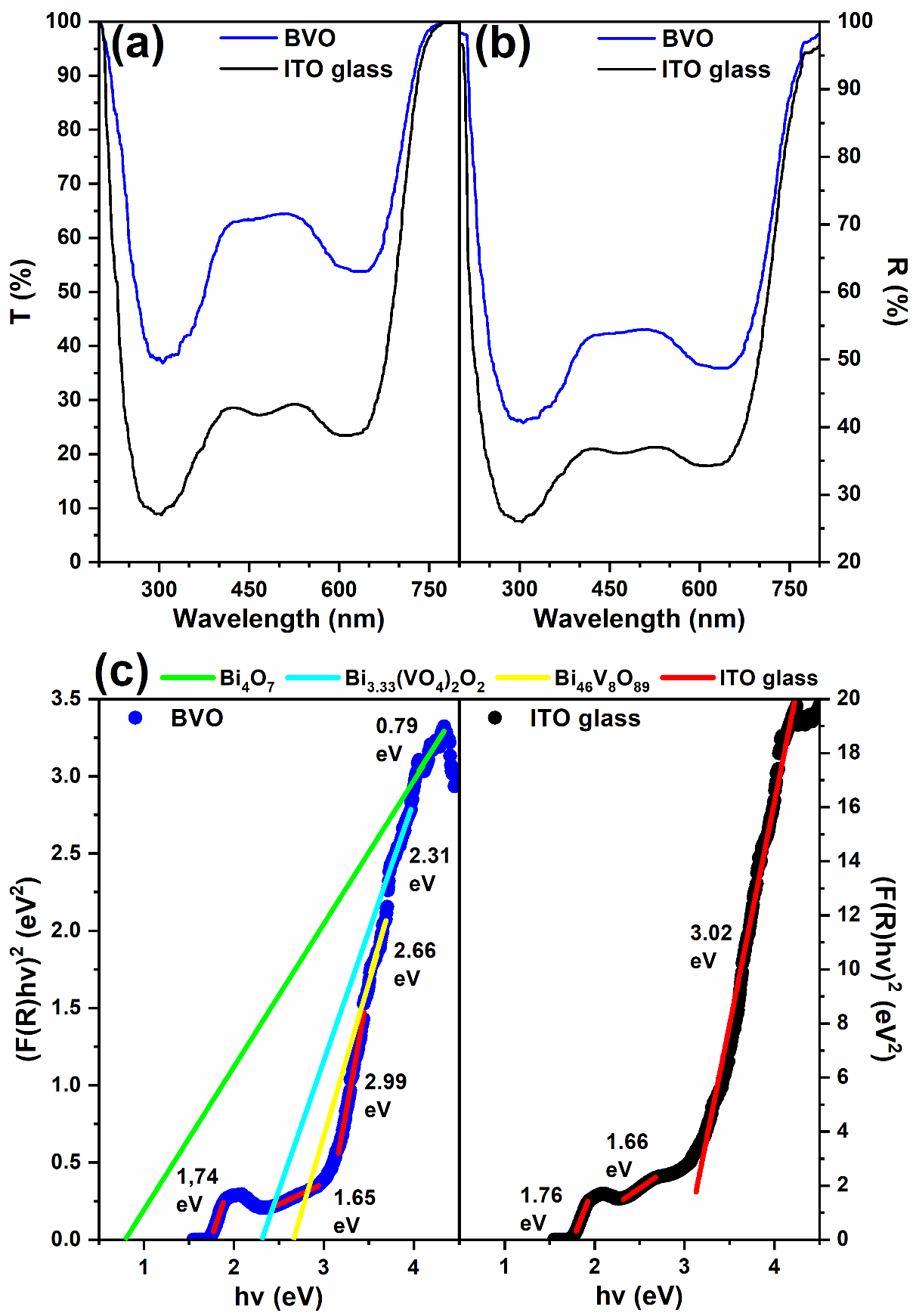


**Figure S3.** (a) Transmittance spectra, (b) Diffuse reflectance spectra and (c) Kubelka–Munk function for estimating and discerning the band–gap energies in the BVO film.


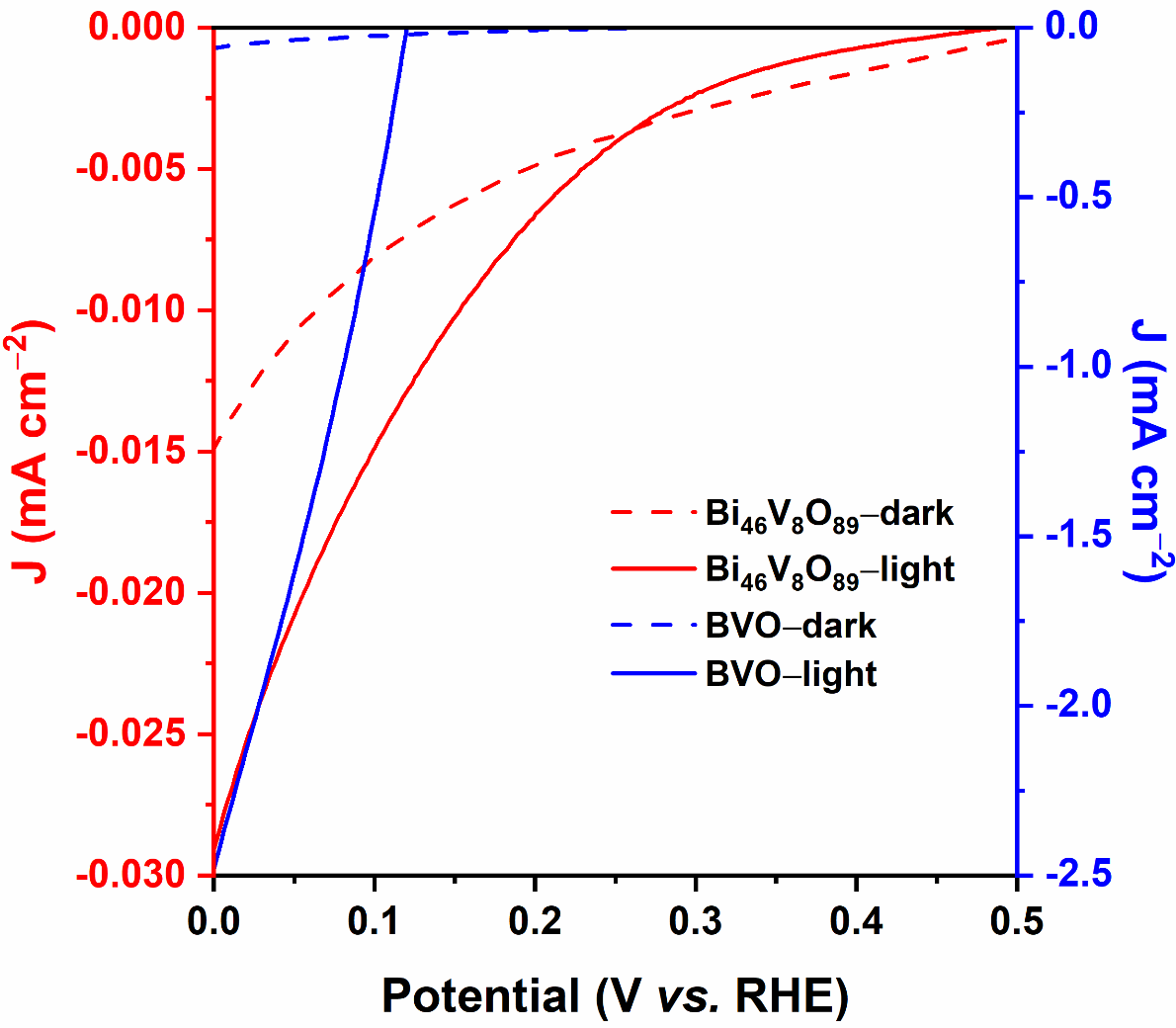


**Figure S4.** Current–potential curves for the prepared BVO film, acting as photocathode. Measurement conditions: active area of 0.2 cm^2^, and 0.5 M Na_2_SO_4_ electrolyte (pH = 6.6). Light Source: Xe Lamp (λ > 450 nm, 100 mW⋅cm^–2^), scan rate of 20 mV⋅s^–1^ from low to high potential, in back illumination mode.


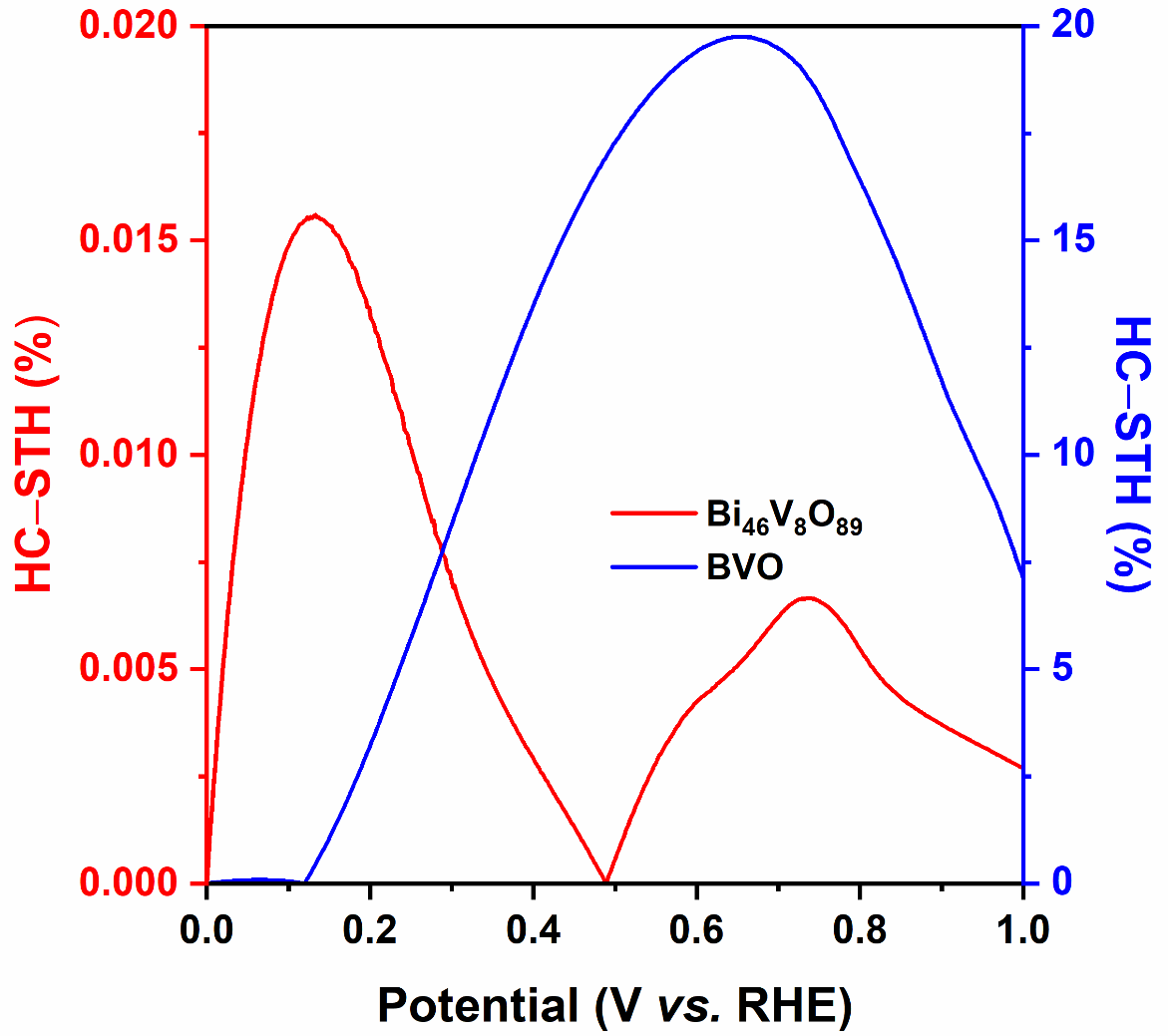


**Figure S5.** Half–cell solar–to–hydrogen (HC–STH) efficiency of the prepared BVO photoelectrode.


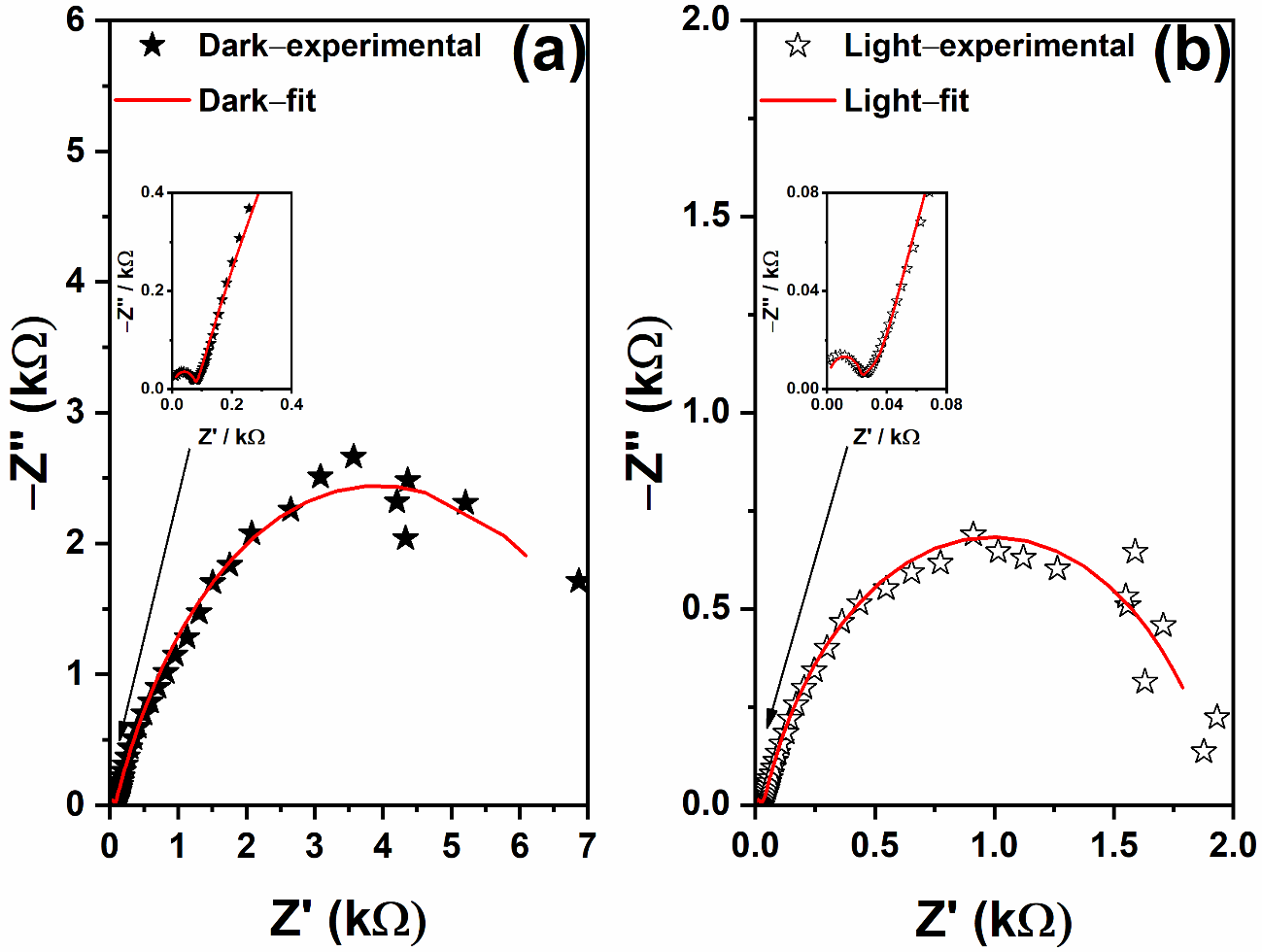


**Figure S6.** Nyquist plots: electrochemical impedance spectra in (a) dark and (b) light of the BVO photoelectrode in a 0.5 M Na_2_SO_4_ electrolyte measured under an applied potential bias of 1.23 V_RHE_ and AC potential of 20 mV. Light Source: Xe Lamp (λ > 450 nm, 100 mW⋅cm^–2^); Frequency range: 100 mHz–100 kHz.


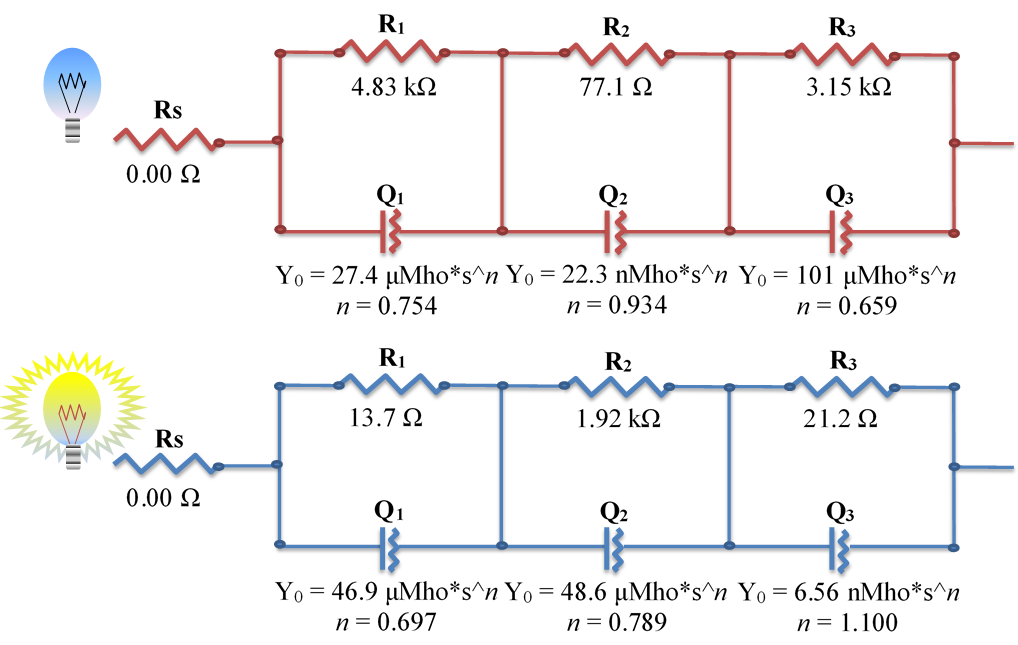


**Figure S7.** Equivalent circuit consisting of three resistance–constant phase elements (Ri–Qi) networks, all connected in series, with each set of R and Q elements linked in parallel, plus a resistance coupled in series (Rs).


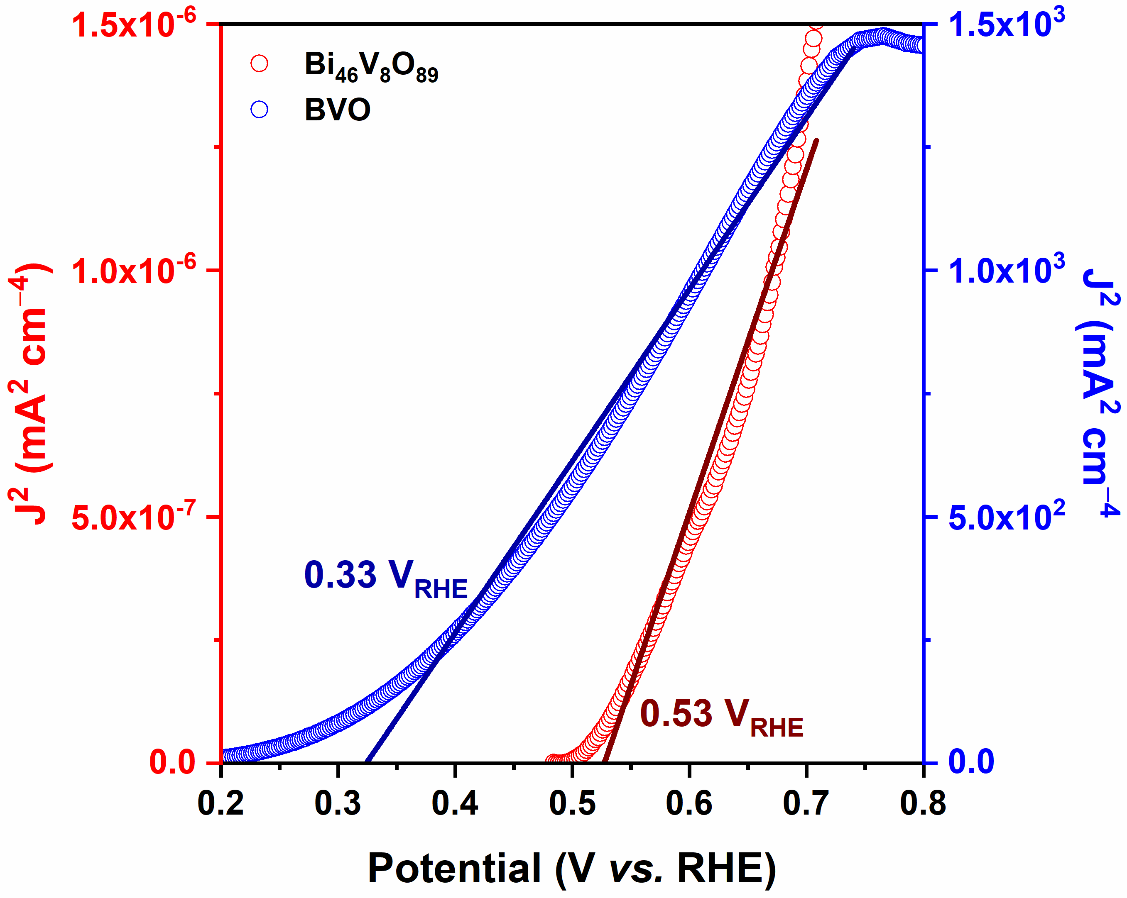


**Figure S8.** Onset potential determination from the J^2^ *vs.* applied potential characteristic for the prepared BVO film while acting as photoanode compared to the film containing only the Bi46V8O89 semiconductor. Light Source: Xe Lamp (λ > 450 nm, 100 mW⋅cm^–2^); electrolyte: 0.5 M Na_2_SO_4_ (pH = 6.6).
